# Repopulation of decellularised porcine pulmonary valves in the right ventricular outflow tract of sheep: Role of macrophages

**DOI:** 10.1177/20417314221102680

**Published:** 2022-06-28

**Authors:** Tayyebeh Vafaee, Fiona Walker, Dan Thomas, João Gabriel Roderjan, Sergio Veiga Lopes, Francisco DA da Costa, Amisha Desai, Paul Rooney, Louise M Jennings, John Fisher, Helen E Berry, Eileen Ingham

**Affiliations:** 1 Institute of Medical and Biological Engineering, School of Biomedical Sciences, Faculty of Biological Sciences, University of Leeds, Leeds, UK; 2 Department of Cardiac Surgery, Santa Casa de Curitiba, Pontifica Universidade Catolica do Parana, Curitiba, Brazil; 3 Institute of Medical and Biological Engineering, School of Mechanical Engineering, University of Leeds, Leeds, UK; 4 NHS Blood and Transplant, Tissue and Eye Services, Estuary Banks, Liverpool, UK

**Keywords:** Decellularised porcine heart valves, cardiac valves, pulmonary roots, macrophages

## Abstract

The primary objective was to evaluate performance of low concentration SDS decellularised porcine pulmonary roots in the right ventricular outflow tract of juvenile sheep. Secondary objectives were to explore the cellular population of the roots over time. Animals were monitored by echocardiography and roots explanted at 1, 3, 6 (*n* = 4) and 12 months (*n* = 8) for gross analysis. Explanted roots were subject to histological, immunohistochemical and quantitative calcium analysis (*n* = 4 at 1, 3 and 12 months) and determination of material properties (*n* = 4; 12 months). Cryopreserved ovine pulmonary root allografts (*n* = 4) implanted for 12 months, and non-implanted cellular ovine roots were analysed for comparative purposes. Decellularised porcine pulmonary roots functioned well and were in very good condition with soft, thin and pliable leaflets. Morphometric analysis showed cellular population by 1 month. However, by 12 months the total number of cells was less than 50% of the total cells in non-implanted native ovine roots. Repopulation of the decellularised porcine tissues with stromal (α-SMA+; vimentin+) and progenitor cells (CD34+; CD271+) appeared to be orchestrated by macrophages (MAC 387+/ CD163^low^ and CD163+/MAC 387−). The calcium content of the decellularised porcine pulmonary root tissues increased over the 12-month period but remained low (except suture points) at 401 ppm (wet weight) or below. The material properties of the decellularised porcine pulmonary root wall were unchanged compared to pre-implantation. There were some changes in the leaflets but importantly, the porcine tissues did not become stiffer. The decellularised porcine pulmonary roots showed good functional performance in vivo and were repopulated with ovine cells of the appropriate phenotype in a process orchestrated by M2 macrophages, highlighting the importance of these cells in the constructive tissue remodelling of cardiac root tissues.

## Introduction

Cardiac valve disease affects all ages. It has a global prevalence and affects all populations. The aortic valve is the most critical and most prone to failure. Cardiac valve replacement surgery is the standard treatment for severe disease and worldwide, approximately 300,000 cardiac valve interventions are performed annually.^1^ Conventional cardiac valve replacements all have limitations. Patients with mechanical heart valves require lifelong anticoagulation therapy.^2^ Bioprosthetic valves suffer from limited durability due to degeneration and calcification restricting their use to the elderly.^3^ For children with congenital heart disease and young adults who are still growing there is currently no ideal valve substitute. The pulmonary autograft switch operation (the Ross procedure) can be performed in this group of patients^4^ in order to provide a living, functional aortic valve. A cryopreserved allograft valve is then placed in the pulmonary position. Allografts fail to repopulate with endogenous cells in vivo, have the potential to initiate immunological graft rejection and cannot grow and develop in the patient. Degeneration of the allograft pulmonary valve leads to reoperation.^4,5^ There is therefore a pressing need for pulmonary valve substitutes which can be used in the Ross procedure, which will grow and develop in the young patient and last a lifetime.

In order to minimise the immune response to cardiac valve allografts, and overcome the limitations of degeneration over time, there has been increasing interest in the use of decellularised pulmonary valve allografts. Clinical studies of the CryoValve SG™ pulmonary valve developed by Cryolife (USA) reported comparable or improved performance compared to cryopreserved pulmonary valve allografts in the short- to medium term (1 to 8 years^6–11^) and in the mid to longer term (5 and 10 years), especially in the paediatric population.^12^ Other groups have used different processes to decellularise pulmonary root allografts, notably the Hannover group^13,14^ and the da Costa group from Brazil and have reported similar short to medium term success with decellularised pulmonary root allografts.^15–17^ A robust systematic review of the literature on the clinical use of decellularised pulmonary valve allografts concluded that decellularised heart valves implanted within the right ventricular outflow tract demonstrated significantly lower reoperation rates when compared to standard tissue conduits.^18^

Decellularised allografts, however, suffer from limitations of availability, particularly in sizes adequate for children. Several groups have therefore investigated the potential use of decellularised porcine valves which could be available ‘off the shelf’ in a range of sizes. Initial clinical use of porcine pulmonary roots prepared using the SynerGraft™ process, however proved disastrous,^19^ due to incomplete decellularisation and the presence of whole porcine cells and residual α-Gal.^20^ The Matrix P^®^ and Matrix P plus^®^ acellular porcine pulmonary valves marketed by AutoTissue GmbH showed good results in pre-clinical and early clinical studies^21,22^ but the subsequent clinical performance of these valves was disappointing^23–26^ with young patients requiring early re-operation (less than 2 years) due to inflammatory and fibrotic responses, believed to be due to incomplete decellularisation and use of a glutaraldehyde fixed cellular skirt upon analysis of pre-implant valves.^24^ It is clearly evident that it is of paramount importance to ensure an absence of cells, DNA and α-Gal from all regions of decellularised porcine valves prior to future consideration of clinical use.

We initially developed proprietary methods for the decellularisation of porcine aortic cardiac valves^27–29^ using low concentration sodium dodecyl sulphate (SDS) and proteinase inhibitors. A similar process has been successfully applied to cryopreserved pulmonary allografts which have been used clinically.^15–17^ We subsequently applied the robust process in the decellularisation of porcine pulmonary roots and showed that cells, and the majority of the DNA, were absent from all tissue regions. Both immunohistochemical labelling and antibody absorption assay confirmed a lack of α-gal epitopes in the decellularised porcine pulmonary root tissue. The biomechanical, hydrodynamic and leaflet kinematics properties were minimally affected by the process.^30^ In a preliminary proof of concept study, decellularised porcine aortic roots were implanted in the pulmonary position in juvenile sheep over a 6-month period.^31^ The study indicated no evidence of a specific immune response. The implanted roots were repopulated with cells, but repopulation was incomplete in the root wall, possibly due to the mismatch in thickness of the aortic root in the pulmonary position or the limited 6-month study period. There was evidence that macrophages were involved in process of cellular repopulation but the longer-term outcome remained unknown.

Studies carried out by the Badylak group have characterised the macrophage response to xenogeneic acellular extracellular matrix scaffolds used in soft tissue repair including porcine small intestinal sub mucosa (SIS ^32^) and porcine urinary bladder.^33^ Studies in rats identified a role for M2 macrophages in constructive tissue remodelling. Indeed, macrophage involvement in the host response to acellular biologic scaffold materials is believed to be necessary for a constructive and functional outcome.^34^ The underlying mechanisms are largely unknown; however, it is recognised that materials such as SIS and urinary bladder used for soft tissue repair must be adequately decellularised and biodegradable otherwise an inflammatory macrophage (M1) response leads to poor outcomes.^35^ There is however a lack of research investigating the role of macrophages in the response to more complex, functional tissue scaffolds such as decellularised cardiac valves. In light of the above, a longer term, temporal study of decellularised valved conduits in sheep was considered important in order to better understand the longer-term functional performance and the role of macrophages in the constructive remodelling process.

The primary objective of this study was therefore to evaluate the functional performance of decellularised porcine pulmonary roots implanted into the right ventricular outflow tract of juvenile sheep over a 12-month period. The secondary objective was to explore cellular repopulation of the decellularised roots in vivo over time. This was carried out using histology and a panel of antibodies including antibodies to CD163 (M2 macrophages), CD80 (M1 macrophages), MAC 387 (recently infiltrating macrophages), progenitor markers (CD34, CD271), stromal cells and lymphocytes. Cryopreserved ovine cellular allografts implanted for 12 months, and non-implanted cryopreserved ovine cellular allografts were evaluated for comparative purposes. The juvenile sheep was selected as the model for the study due to similar valve biomechanics and haemodynamics to the human.^36^ The juvenile sheep implant model is used in cardiac valve research because it is an excellent predictor of the durability and performance of biologic heart valves as affected by calcification.^37^

## Materials and methods

### Porcine and ovine pulmonary roots

Porcine pulmonary roots of 18–20 mm diameter were harvested from the hearts of Large White pigs (50–55 kg) and ovine pulmonary roots from 15-month-old sheep (15 mm; Texel) sourced from a UK licenced abattoir within 3–4 h of slaughter. The pulmonary roots were dissected, and the valve diameter measured using cylindrical sizing instruments. The roots were trimmed of excess myocardium and connective tissue, rinsed in phosphate buffered saline (PBS; Oxoid). Cellular porcine and cellular ovine pulmonary roots were cryopreserved (see below). Porcine roots for decellularization were stored dry on PBS moistened Whatman No. 1 filtre paper, at −20°C.

### Decellularisation

Porcine pulmonary roots were decellularised in five batches of eight valves as described previously^30^ using aseptic technique. Roots were thawed at 37°C for 30 min and the myocardium trimmed to 10 mm length and 3–4 mm thickness. The adventitia of the pulmonary artery wall was scraped three to four times with a scalpel blade. Roots were incubated in 200 ml PBS containing 0.05 mg ml^−1^ vancomycin (Merck), 0.5 mg ml^−1^ gentamicin (Merck) and 0.2 mg ml^−1^ Polymixin B (Merck) for 16–17 h at 4°C. The valve leaflets were protected by cotton wool soaked with 50% (v/v) foetal calf serum (FBS; Biosera) in PBS and the adventitia of the pulmonary wall and myocardium skirt were treated with trypsin (0.5% (w/v) agarose gel; 1.125 × 10^4^ U ml^−1^ trypsin type II-S from porcine pancreas, Sigma) using a small Artist’s paint brush, placed in a humidified container and incubated for 2 h at 37°C. Roots were washed (3 × 30 min) in PBS containing 0.1% (w/v) EDTA (Sigma), 5 × 10^3^U ml^−1^ trypsin inhibitor (Sigma) and 10 kIU ml^−1^ aprotinin (Mayfair House) at 42°C with agitation on an orbital shaker (130 rpm). Roots were transferred into hypotonic tris buffer (10 mM tris (Sigma), pH 8.0 containing 0.1% (w/v) EDTA plus 10 kIU ml^−1^ aprotinin) and incubated for 24 h at 42°C with agitation (130 rpm). The roots were incubated in 0.1% (w/v) sodium dodecyl sulphate (UltraPure™ SDS solution; Gibco) in hypotonic tris buffer (10 mM tris, pH 8.0 containing 0.1% (w/v) EDTA plus 10 kIU ml^−1^ aprotinin) for 24 h at 42°C (130 rpm). The roots were washed (2 × 30 min, 1 × 16–17 h) with PBS containing aprotinin (10 kIU ml^−1^) at 42°C (130 rpm) and incubated in nuclease solution (Benzonase 10 U ml^−1^ (Novagen) for 2 × 3 h at 37°C and 80 rpm). This was followed by washing (3 × 30 min) in PBS followed by washing in hypertonic buffer (50 mM tris buffer pH 7.5–7.7, plus 1.5 M NaCl) for 24 h at 42°C (130 rpm). Following washing in PBS (2 × 30 min and 2 × 24 h), the roots were immersed in peracetic acid (0.1% w/v; Sigma) for 4 h at 37°C and washed in PBS at 42°C (3 × 30 min, 1 × 60–66 h) prior to either analysis or cryopreservation.

Two roots from batches 1 to 4 were analysed using histology, determination of total DNA content and sterility for quality assurance analysis (QA) and the remaining six roots from batches 1 to 4 were cryopreserved for implantation in juvenile sheep (24). Batch 5 roots were cryopreserved for subsequent biomechanical analysis.

### Cryopreservation of pulmonary roots

Each root was placed into a nylon bag in 100 ml cryomedium (HBSS; 16% v/v DMSO, 25 mM HEPES; product code 04-311, Inverclyde Biologicals). The bag was heat-sealed and placed into a foil bag that was also heat-sealed. The foil bag was then placed into a Jiffy bag before freezing and storage at −80°C.

### Quality assurance (QA) analysis of decellularised porcine pulmonary roots

**Sterility:** The final PBS wash solution (100 µl) from decellularisation of all porcine pulmonary roots was streaked onto nutrient agar, fresh blood agar, heated blood agar and Sabouraud (SAB) dextrose agar (all Oxoid). A sample of the distal pulmonary wall from QA pulmonary roots (3 × 5–7 mm) was aseptically transferred to 10 ml of nutrient broth. The plates and nutrient broth were incubated at 37°C for 48–72 h (30°C for 5 days for SAB agar).

**Histology:** Longitudinal samples of each QA root incorporating half a leaflet, the leaflet insertion (junction), pulmonary artery wall and myocardial skirt were fixed in 10% (v/v) neutral buffered formalin (NBF), dehydrated and embedded in paraffin wax. Longitudinal serial sections (10 µm) were cut at two levels 120 µm apart. Sections from each level were stained with haematoxylin and eosin (H&E) and DAPI using standard methods.^30^

**Total DNA content:** DNA was extracted from 90 to 300 mg wet weight of the pulmonary wall, junction, ventricular muscle and the remaining two and half leaflets of the QA roots using the DNeasy Blood & Tissue Kit (Qiagen). The concentration of DNA in the extracts was determined by Nanodrop spectrophotometry at 260 nm. In parallel, DNA was extracted and quantified from triplicate samples of native porcine pulmonary root wall, leaflet, junction and ventricular muscle (24–28 mg) for comparative purposes.

### In vivo performance of decellularised porcine pulmonary valves

**Study design:** The in vivo study was conducted at the University Veterinary Hospital ‘Hospital Veterinário Para Animais De Companhia’ PUC-PR São José Dos Pinhais, Brazil. The study was performed in accordance with NIH guidelines and the Institutional guidelines for animal care and were approved by the Ethical Committee of Researches PUC-PR (project approval number CEUA 511). Sheep were 120 days old (male and female) Texel breed. Twenty-two sheep were implanted with cryopreserved decellularised porcine pulmonary roots in the right ventricular outflow tract for macroscopic and biological evaluation at 1, 3, 6 and 12 months (four per time point) and at 12 months (n = 4) for evaluation of material properties (Leeds). A further four sheep were implanted with cryopreserved cellular ovine pulmonary allografts, Santa Ines breed, males, 150 days old and harvested at 12 months for comparative evaluation of the biological performance. We did not include groups of ovine allografts for comparison of biological outcomes at 1, 3 and 6 months or material properties at 12 months since our previous studies of allogenic ovine aortic roots^31^ in the RVOT of juvenile sheep had shown complete calcification after 6 months implantation.

The material properties of the decellularised porcine pulmonary roots following 12 months implantation in sheep (which had been cryopreserved prior to implantation) were compared to non-implanted cryopreserved decellularised porcine pulmonary roots and to non-implanted cryopreserved native (cellular) ovine pulmonary roots. The effect of cryopreservation on the material properties of cellular porcine pulmonary roots and effect of decellularisation followed by cryopreservation on the material properties of porcine pulmonary roots was also evaluated.

**Surgical procedure and animal husbandry:** Cryopreserved decellularised porcine pulmonary roots were shipped on dry ice to PUC-PR through a courier with appropriate import licences in place. They were stored at −80°C until implanted. Ovine allograft roots for the in vivo study were cryopreserved as described above. The cryopreserved roots were gently thawed at 37°C, washed aseptically in 0.9% (w/v) saline solution, trimmed and kept moist until implanted.

Sheep were brought to the veterinary hospital 48 h before the surgical procedure, fasted and housed in a purpose-built facility. Sheep were weighed and administered diazepam 0.5 mg kg^−1^ and butorphenol 0.4 mg kg^−1^ through an intravenous line and placed in lateral recumbency. Arterial pressure was monitored via direct arterial line in the radial artery. General anaesthesia was induced using Propofol at 4 mg kg^−1^ and maintained using Propofol 0.5 mg kg^−1^ min^−1^. Endotracheal intubation was performed, and mechanical ventilation established. Arterial blood gases were monitored throughout. A left lateral thoracotomy was performed through the third intercostal space following Bupivacaine injection. The pericardium was opened, and the heart and great vessels identified. Heparin 150 U kg^−1^ was given intravenously. Cardiopulmonary bypass was established through cannulation of the descending aorta and the right atrium, the pulmonary artery was dissected and clamped proximally and distally just before the bifurcation. A segment of the pulmonary artery was resected, and the pulmonary valve leaflets were removed. The decellularised porcine or ovine allogeneic pulmonary root was then anastomosed using 5/0 Prolene continuous suture proximally and distally. The clamps were removed, and the function of the pulmonary root implant was observed.

Cardiopulmonary bypass was weaned, and the wound was closed in three layers using Vicryl absorbable material. The animals were allowed to surface from the anaesthesia, monitored for heart rate, respiratory rate and blood pressure continuously. Animals were observed for their clinical behaviour, specially looking for any sign of discomfort and non-steroidal analgesia was administered intravenously if required. Once the anaesthetist had confirmed adequate postoperative status, the sheep was extubated, the arterial line removed and the sheep was then moved to a recovery room, where the animal continued under close observation for 2 h. After 24–48 h, sheep were housed in covered open air pens where water was freely available. Sheep received prophylactic gentamicin 4 mg kg^−1^ and cephalosporin 2 mg kg^−1^ until the fifth postoperative day.

**Clinical follow-up:** The general health of the sheep was monitored by a veterinary surgeon twice weekly throughout the in life-phase. The animals were weighed at the time of implantation and then at 1, 3, 6 and 12 months (for surviving animals). The animals were monitored by Doppler echocardiography at 1, 3, 6 and 12 months (for surviving animals). Images were captured and observations made were of overall valve function, calcification, leaflet thickness and stenosis and any valve insufficiency. The diameter of the annulus, sinus and conduit were recorded. The Doppler study allowed measurements of the velocity of blood passing through the valves; the velocity of the blood was then used to calculate the pressure gradient according to the ‘Bernoulli equation’. Implanted valves were retrieved by performing a redo left thoracotomy (as above) under general anaesthesia and the animals were sacrificed (KCl 19.1% iv) without recovery. The implanted pulmonary roots were placed in saline solution 0.9% (w/v), washed, subject to macroscopic analysis and processed for histology/immunohistochemistry/calcium analysis as described below or cryopreserved.

**Gross analysis of explanted pulmonary roots:** The explanted roots were surveyed macroscopically and photographed. Gross analysis of the explanted valves included observations of calcification, leaflet retraction, fenestrations, thrombi and vegetations. The presence of any of these features was scored on a scale of 0–3 where 0 was none, 1 was minimal, 2 was moderate and 3 was severe/complete.

### Processing of pulmonary roots for histology, immunohistochemistry and calcium analysis

Non-implanted ovine pulmonary roots (*n* = 4), the decellularised porcine pulmonary roots explanted at 1, 3, 6 and 12 months (*n* = 4; *n* = 2 for 6 months) and ovine allograft roots explanted at 12 months (*n* = 4) were dissected and fixed using the same process. Each pulmonary root was dissected longitudinally into three samples, each comprising ventricular muscle, proximal suture line, junction, leaflet pulmonary wall and distal suture line. Two samples were fixed in 25 ml 10% (v/v) NBF for 24 h. One sample was fixed in zinc fixative (0.1 M tris; 3.2 mM calcium acetate (Thermo Fisher), 27 mM zinc acetate (Sigma), 37 mM zinc chloride (Fluka); pH 7.2) for 24 h. The samples were transferred to 25 ml 70% (v/v) ethanol in labelled pots. Four decellularised porcine pulmonary roots explanted at 12 months were cryopreserved (as above) and stored at −80°C. The explanted roots were shipped to Leeds in 70% (v/v) ethanol at ambient temperature or on dry ice (cryopreserved roots) by courier with appropriate export (Ministerio da Fazenda, Brazil) and import (DEFRA) licences. One NBF fixed sample of each pulmonary root was used for histological and immunohistochemical analysis and one for quantitative calcium analysis. Zinc-fixed samples were used for immunohistochemistry with antibodies which were ineffective on NBF-fixed tissues.

### Histological and immunohistochemical analysis of explanted decellularised porcine, explanted ovine and non-implanted ovine pulmonary roots

NBF and zinc fixed samples were dissected longitudinally into two portions. Each portion was dissected horizontally through the mid-part of the pulmonary artery wall into proximal and distal portions. The proximal and distal portions of each half were placed together in a histocassette, dehydrated and embedded in paraffin wax using a Leica automatic tissue processor. Embedded samples were serially sectioned (8 µm) using a microtome. The first 25 sections were retained (level 1), 100 sections discarded, 50 sections retained (level 2), 75 sections discarded and the last 25 sections retained (level 3). One section from each level of each root was stained using H&E, Sirius Red Miller`s, Masson`s trichrome, von Kossa and DAPI using standard protocols.

Sections of the pulmonary root tissues were stained with a range of antibodies selected to determine the phenotype of the cells present within the tissues (Table 1). The primary antibodies, dilutions, method of antigen retrieval and positive control tissues were established in extensive preliminary studies and details of the protocols are summarised in Table 1. Isotype control antibodies and omission of the primary antibodies were used to verify antibody specificity and as negative controls using sections from level 2. All images were captured using an upright Carl Zeiss Axio Imager.M2 incorporating an Axio Cam MRc5, which was controlled by Zen Pro software 2012 (Zeiss).

**Table 1.** Antibodies and methods used for detection of cells in pulmonary root tissues.

Antibody	Source/dilution	Antigen retrieval	Detection	Control	Isotype	Fixation
α-SMA Smooth muscle cells	DAKO M0851 IgG2a; 1/100	Tris/ EDTA^a^	Thermo Ultravision	Sheep pulmonary artery	DAKO X0943 IgG2a; 1/140	NBF
Vimentin	Leica NCL-L-VIM-V9 IgG1; 1/800	None	DAKO Anti-mouse HRP	Sheep skin	DAKO X0931 IgG1; 1/5000	Zinc
CD3 T-cells	Novacastra NCL-L-CD3-565 IgG1; 1/125	None	Thermo Ultravision	Sheep thymus	DAKO X0931 IgG1; 1/312	Zinc
Ki67 Proliferating cells	DAKO M7240 IgG1;1/150	Citrate^b^	Thermo Ultravision	Sheep small intestine	DAKO X0931 IgG1; 1/333	NBF
CD271 Progenitor cells	Biolegend ME20.4 IgG1; 1/100	Proteinase K^c^	DAKO Envision	Sheep carotid artery	DAKO 0931 IgG1; 1/10	NBF
CTGF Connective tissue GF	Abcam Ab6992 Rabbit; 1/100	Citrate	Thermo Ultravision	Sheep skin	DAKO X0936 Rabbit Ig; 1/1500	NBF
CD19 B-cells	Novacastra NCL-L-CD19-163 IgG2b; 1/50	None	DAKO Envision	Sheep lymph node	DAKO IgG2b; 1/143	Zinc
MAC 387 Macrophages	AbD Serotec MCA874GA IgG1; 1/100	Proteinase K	DAKO Envision	Sheep carotid artery	DAKO X0931 IgG1; 1/10	NBF
VWF Endothelial cells	DAKO A0082 Rabbit; 1/200	Proteinase K	DAKO Envision	Sheep carotid artery	DAKO X0936 Rabbit Ig; 1/1500	NBF
CD163 M2- macrophages	AbD Serotec MCA 1853 IgG1; 1/100	Proteinase K	DAKO Envision	Sheep lymph node	DAKO X0931 IgG1; 1/10	NBF
CD80 M1- macrophages	AbD Serotec MCA 2436GA IgG1; 1/25	Proteinase K	DAKO Envision	Sheep lymph node	DAKO X0931 IgG1; 1/2.5	NBF
CD34 Progenitor cells	AbcamAb81289 EP373Y IgG Rabbit Mab; 1/500	Tris microwave^d^	DAKO Envision	Sheep lung	DAKO X0936 Rabbit IgG; 1/14,200	NBF

NBF: neutral buffered formalin.

a Tris/EDTA buffer (10 mM Tris, 1 mM EDTA, pH 9.0) microwave high power 10 min.

b Citrate buffer (10 mM, pH 6.0) microwave high power 10 min.

c Proteinase K (DAKO S302080) 20 min at room temperature.

d Tris buffer (10 mM, pH 9.0) microwave high power 10 min.

### Cell counting

For sections stained with DAPI and antibodies to MAC 387, CD163 and CD80 sections from each level were subject to cell counting. For all other antibody labelling, cells were counted using sections from level 2. Eleven fields of view (FoV; 100× magnification) were identified in each section using a pre-determined template representing the adventitia, media and intimal regions of the distal, mid and proximal pulmonary artery wall, proximal and distal leaflet. The total number of cells in the DAPI stained sections and all the cells expressing a given marker within each FoV was counted using Image J software. The area of a field of view was 0.576 mm^2^. The mean number of cells per FoV were then multiplied by 1.74 to give the mean number of cells per mm^2^. The mean number of cells in the adventitia, media and intimal region of the pulmonary artery wall and leaflet for each pulmonary root was then calculated. The mean total number of cells expressing each marker in each region was then divided by the total number of cells in each region to calculate the percentage of cells expressing the marker.

Statistical analysis of the morphometrical data was undertaken in Minitab 18. The total number of cells per mm^2^ and the total number of cells expressing a given marker per mm^2^ within each region of the valved conduits for each group was compared using Welch’s ANOVA (equal variances not assumed) since the data had unequal variances. Games-Howell pairwise comparisons were then applied to determine individual differences (*p* < 0.05) between group means. Percentage data (percent of cells expressing a given marker) was arc sin transformed in order to normalise the data. The group means and 95% confidence limits were calculated using the arc sin transformed data. The means, upper and lower 95% confidence limits were then back-transformed to percentage data for presentation purposes. The transformed data had unequal variances. The arc sin transformed data was analysed using Welch`s ANOVA. Games-Howell pairwise comparisons were then applied to determine individual differences (*p* < 0.05) between group means.

### Quantitative calcium analysis

NBF fixed samples of the non-implanted ovine pulmonary roots (*n* = 4), the explanted decellularised porcine pulmonary roots, ovine allograft explanted roots and non-implanted decellularised porcine pulmonary roots (*n* = 4) were analysed for calcium content. The tissues were divided into five samples of 50–300 mg wet weight corresponding to the (1) distal suture line, (2) distal pulmonary wall, (3) proximal pulmonary wall, (4) proximal suture line and (5) leaflet. The samples were blinded and sent to ALS Scandinavia AB, Aurorum 10, 977 75 Luleå, Sweden for calcium analysis. Briefly, tissues were weighed and then immersed in 5 ml HNO_3_ and digested at 600 W power for 60 min before being analysed using high-resolution inductively coupled plasma mass spectrometry (ICMPS). Data was returned as mg calcium per kg (wet weight) of tissue (ppm). Data was analysed in Microsoft Excel 13 by one-way analysis of variance followed by calculation of the MSD (*p* < 0.05) using the T-method, comparing the levels of calcium within a given tissue site between all groups of pulmonary roots.

### Biomechanical evaluation of ovine and porcine pulmonary root tissues

The following groups of pulmonary roots were subjected to uniaxial tensile testing: cellular porcine (Nat porcine; *n* = 6), cryopreserved cellular porcine (Nat C porcine; *n* = 6), cryopreserved decellularised porcine (Decell C porcine; *n* = 6), cryopreserved cellular non-implanted ovine (Nat C ovine; *n* = 6) and 12-month explanted decellularised porcine (12 m Decell C porcine; *n* = 4). All cryopreserved roots were thawed on the day before testing and stored at 4°C in 100 ml Cambridge antibiotic solution (Source BioScience). Before testing, the roots were rinsed with PBS and sizes of the roots (internal diameter) were measured with cylindrical sizing instruments.

In order to compare the material properties of pulmonary root tissues, a single ramped uniaxial tensile test to failure was performed. A materials testing machine (3365K5747, Instron^®^ Corporation; High Wycombe, Buckinghamshire, UK) fitted with a 50 N Load cell and BioPuls Bath was employed for all the tensile tests. Tissue specimens with 10 mm gauge length and 5 mm width were prepared from the root wall in the axial and circumferential directions from each root, and from the valve leaflet in the circumferential direction. Due to size limitations, the radial specimens from valve leaflets were 3 mm in width with a 6 mm gauge length. Each tissue specimens’ thickness was measured with a digital thickness gauge J-40 V; having a precision of 0.01 mm. The wall and leaflet specimens of each root were then subject to uniaxial tensile testing to failure at 10 mm min^−1^ strain rate, similar to previous studies.^30,38,39^ In brief, specimens were mounted in their relaxed state, within the BioPuls Bath containing PBS at 37°C. The specimens were then loaded to failure using a positive ramp function at a rate of 10 mm min^−1^ and the load-elongation response recorded. During testing, load data from the load cell and extension data from the stroke of the tensile machine was acquired, which was then converted into stress-strain data from which the ultimate tensile stress (UTS) and stiffness (elastin phase slope and collagen phase slope) were determined.

Data was analysed by one-way analysis of variance (ANOVA; SPSS statistics software for Windows, Version 21.0). Gabriel post hoc analysis was used to determine the significances between individual group means (*p* < 0.05).

## Results

### Quality assurance of decellularised porcine pulmonary valves for implantation

**Figure 1.**
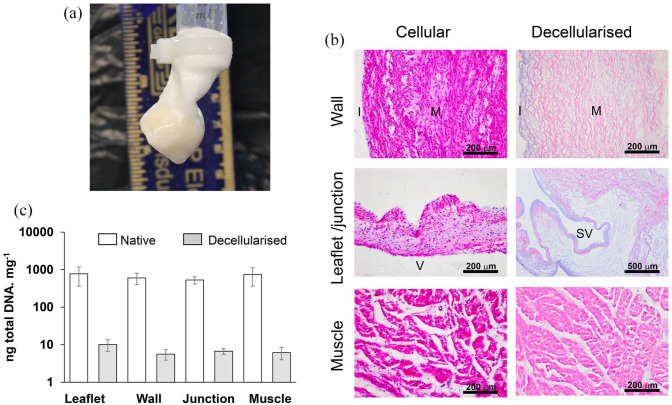
Efficacy of decellularisation of porcine pulmonary roots: (a) Image of decellularised porcine pulmonary root undergoing competency testing, (b) representative images of sections of cellular and decellularised porcine pulmonary root tissues stained with H&E. Images captured at 10× magnification (scale bars 200 μm) except image of decellularised junctional region (4× magnification, scale bar 500 μm). I: intimal region; M: medial region; SV: sinus of Valsalva; V: ventricularis. (c) Total DNA content of different tissue regions of native and decellularised porcine pulmonary roots. Data is expressed as the mean (n = 3 for native; n = 8 for decellularised) ng DNA mg^−1^ (wet weight) ± 95% confidence limits.

Sterility testing of the 8 QA decellularised porcine pulmonary roots showed no evidence of microbial growth on any microbiological media. An image of a decellularised root is presented in Figure 1(a). Histological evaluation of the 8 QA roots revealed no evidence of cells (H&E) or cell nuclei (DAPI) in any of the different regions (leaflet, pulmonary artery wall, junction and ventricular muscle; Figure 1(b)). There was less than 18 ng DNA per mg in all tissue regions for all eight decellularised roots (Figure 1(c)). The overall reduction in total DNA content in the decellularised tissues compared to native porcine pulmonary root tissues was greater than 97.5%.

### Post-operative monitoring of animals and clinical observations

Two sheep were lost during the immediate post-operative period. One sheep suffered a cardiac arrest, and one sheep was lost due to pneumothorax. These sheep were replaced. A further sheep from the 6-month group died at 6 months. The root was explanted and found to have vegetations covering the whole of the root, indicating that the likely cause of death was endocarditis. The remaining sheep all remained clinically healthy throughout the follow-up period. The weights of the sheep in all groups steadily increased during the study period (Table 2). Sheep that had been implanted with the ovine pulmonary allografts were heavier than the sheep implanted with the decellularised porcine pulmonary roots at the time of implantation and at each time point throughout the study.

**Table 2.** Summary of measurements and observations from Doppler echocardiographic evaluation of the implanted decellularised porcine and cryopreserved ovine pulmonary roots in situ during the course of the study.

	1 month, *n* = 4		3 months, *n* = 4			6 months, *n* = 4 (*n* = 3 at 6 months*)				12 months, *n* = 8					Ovine allografts, *n* = 4				
	0 m	1 m	0 m	1 m	3 m	0 m	1 m	3 m	6 m	0 m	1 m	3 m	6 m	12 m	0 m	1 m	3 m	6 m	12 m
Weight (kg)	21.9 ± 2.8	25.6 ± 4.2	21.8 ± 6.9	25.3 ± 8.8	28.4 ± 4.1	21.6 ± 5.5	26.3 ± 8.6	31 ± 7.5^a^	36 ± 17^a,b^	25.5 ± 1.6	32.2 ± 1.4^a^	34.8 ± 1.9^a^	38.6 ± 2.4^a,b,c^	52.3 ± 2.3^a,b,c^	37.8 ± 4.9	41.7 ± 6.3	57 ± 4.5^a,b^	59.9 ± 0.4^a,b^	71.1 ± 9.6^a,b,c,d^
Diameter	18.8 ± 0.8	18.8 ± 0.8	18.1 ± 1	19 ± 1.3	20.3 ± 3.3	18.3 ± 0.8	21.5 ± 4	21.8 ± 4.6	21.3 ± 2.7	18.1 ± 0.5	20.5 ± 1.7^a^	22.4 ± 1.9^a^	22.4 ± 2.4^a^	21.8 ± 2.1^a^	19.8 ± 2.4	19.8 ± 2.4	21.8 ± 3.5	24.5 ± 2.1^a,b^	24.8 ± 5.7^a,b^
Velocity (mm sec^−1^)	ND	695 ± 304	ND	763 ± 183	745 ± 286	ND	703 ± 435	623 ± 330	577 ± 112	ND	659 ± 125	751 ± 148	646 ± 90	812 ± 241	ND	750 ± 298	1025 ± 571	875 ± 280	695 ± 257
Gradient (mmHg)	ND	2.4 ± 2.2	ND	2.8 ± 1.2	2.8 ± 1.9	ND	2.7 ± 2.1	2.7 ± 2.8	1.6 ± 0.8	ND	2.2 ± 0.7	2.3 ± 0.8	2.1 ± 0.7	3.3 ± 2.2	ND	2.8 ± 2.2	5.1 ± 4.8	4.0 ± 3.0	2.2 ± 1.5
Function	ND	Norm	ND	Norm	Norm	ND	Norm	Norm	Norm	ND	Norm	Norm	Norm	Norm	ND	Norm	Norm	Norm	Norm
Stenosis	ND	Abs	ND	Abs	Abs	ND	Abs	Abs	Abs 2 Alt 1+	ND	Abs	Abs	Abs	Abs	ND	Abs	Abs	Abs	Abs
Insufficiency	ND	Abs 2 Triv 1 Low 1	ND	Abs 2 Triv 1 Low 1	Triv 3 Low 1	ND	Abs 3 Low 1	Abs 2 Low 1 Mod 1	Triv 1 Low 1 Mod 1+	ND	Abs 6 Triv 2	Abs 3 Triv 3 Low 2	Abs 4 Triv 3 Low 1	Abs 5 Triv 2 Low 1	ND	Abs	Abs 2 Triv 2	Abs 2 Low 2	Triv 2 Low 2
Calcification	ND	Abs	Abs	Abs	Abs	ND	Abs	Abs 3 Pos 1	Abs	ND	Abs	Abs	Abs	Abs	ND	Abs	Abs	Abs	Abs
Leaflet mobility	ND	Norm	ND	Norm	Norm	ND	Norm	Norm	Norm 2 Red 1+	ND	Norm	Norm	Norm	Norm	ND	Norm	Norm	Norm	Norm

Abs: No signs of stenosis, insufficiency or calcification; Alt: altered; Mod: moderate; ND: not done; Norm: normal function/mobility; Pos: positive; Red: reduced; Triv: trivial.

Quantitative data was analysed by one-way analysis of variance for each group. Following ANOVA, the minimum significant difference (*p* < 0.05) was determined using the T-method.

a Significantly different than same group at 0 month.

b Significantly different than same group at 1 month.

c Significantly different than same group at 3 months.

d Significantly different than same group at 6 months.

* One sheep in the 6 months group was lost due to endocarditis at 6 months. +A second sheep was also found to have endocarditis at sacrifice at 6 months.

### Doppler echocardiography, valve diameters, velocities and gradients

Observations of valve function, presence of stenosis, valve sufficiency, calcification and leaflet mobility are shown in Table 2. The decellularised porcine and ovine control pulmonary roots were functioning normally in all animals except for two roots explanted at 6 months. The sheep that died at 6 months (endocarditis) showed moderate insufficiency and calcification of the leaflets at 3 months. The second sheep showed severe stenosis and abnormal function with reduced leaflet mobility at the 6-month time point (upon explantation the root from this sheep was also found to have vegetations indicative of endocarditis). None of the other decellularised or ovine pulmonary roots showed evidence of calcification throughout the study and valve insufficiency was absent, low or trivial at all time-points. The data for the diameter of the valves at the annulus for each group at 1, 3, 6 and 12 months are presented in Table 2 together with the data for the diameters of the decellularised porcine pulmonary valves at implantation. The diameters at the annulus increased over time. The recorded velocities of blood flow across the valves were all within the normal range (Table 2) with no significant variation within each group over time (one-way ANOVA). The pressure gradients at all-time points were low, and there was no significant variation within each group over time (one way ANOVA).

### Macroscopic analysis

**Figure 2.**
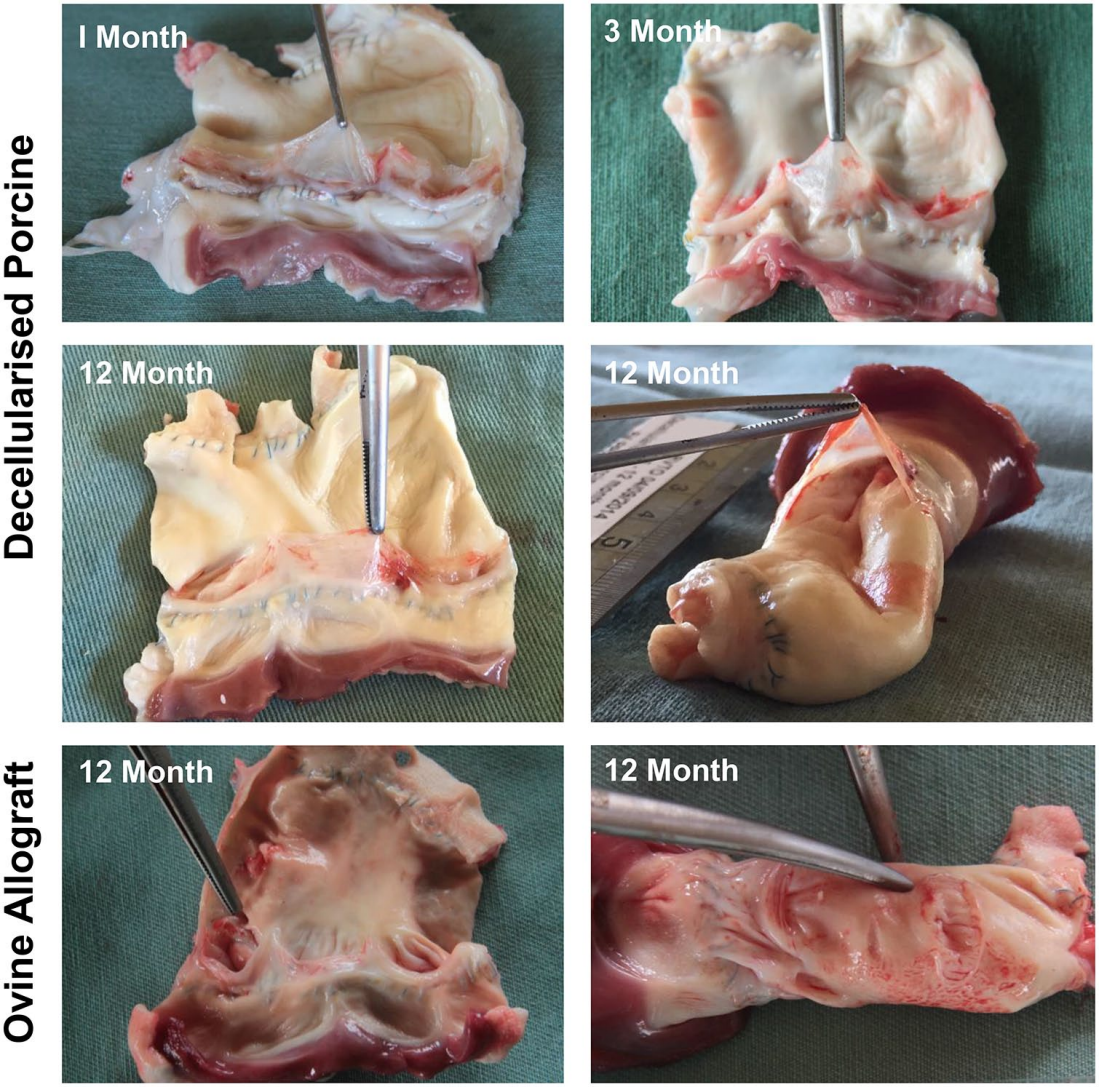
Macroscopic images of explanted pulmonary roots. Images show the exposed leaflets from cut open decellularised porcine pulmonary roots explanted at 1, 3 and 12 months and everted root at 12 months. These images show that the leaflets are thin and translucent with no evidence of thickening, calcification or damage. Images in the bottom row of explanted ovine allografts at 12 months showing aneurism in sinus of Valsalva (left) and rupture in pulmonary artery wall (right). Features are indicated by use of forceps.

Decellularised porcine pulmonary roots explanted at 1 and 3 months showed no evidence of calcification, leaflet retraction, thrombi or vegetations. The leaflets were translucent, thin and had no tears (Figure 2), however one root from each group had a fenestration in one of the leaflets. Two of the decellularised porcine pulmonary roots explanted at 6 months had vegetations (see above). The remaining two 6-month explants showed no overt signs of calcification, cusp retraction, fenestrations, thrombi or vegetations. Two of the decellularised porcine pulmonary roots explanted at 12 months had fenestrations in one leaflet, otherwise the leaflets were thin and translucent (Figure 2). One root had signs of focal calcification in the pulmonary artery wall. There were no other signs of abnormalities in the 12-month explanted decellularised porcine pulmonary roots. None of the explanted ovine allografts (*n* = 4) showed any signs of calcification, cusp retraction, thrombi, fenestrations or vegetations. However, one root had an aneurysm in the sinus of Valsalva (Figure 2), and a second root had a rupture in the pulmonary artery wall (Figure 2).

### Histological evaluation of explanted decellularised porcine pulmonary roots, explanted ovine allografts and non-implanted ovine pulmonary roots

**Figure 3.**
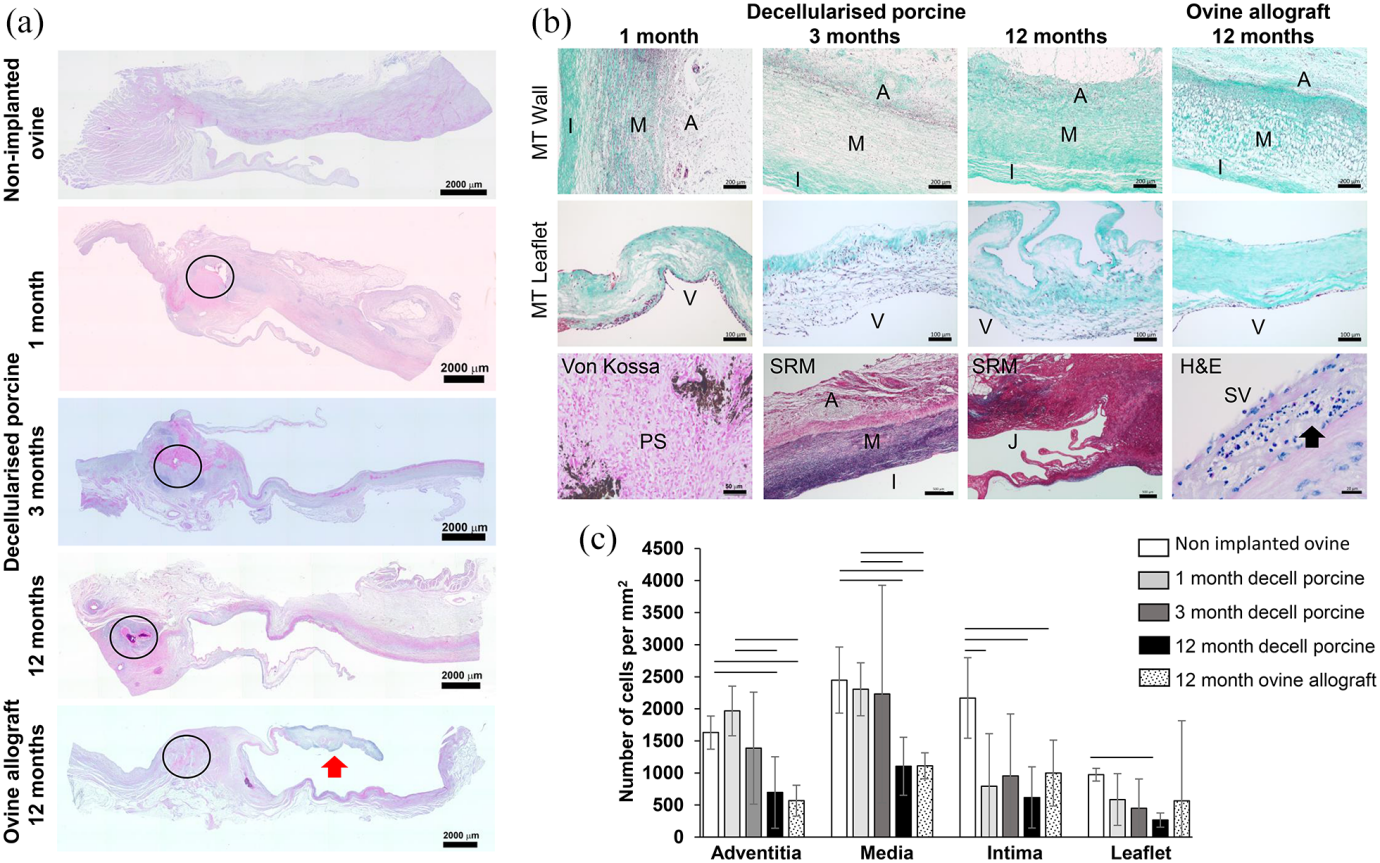
Histological analysis and total number of cells in different regions of native ovine pulmonary roots, explanted decellularised porcine pulmonary roots and ovine allografts. (a) Scanned images of longitudinal sections of the proximal regions of the explanted pulmonary roots compared to a non-implanted ovine root stained with H&E (captured at 2.5× magnification; scale bars 2000 μm). Circles: proximal suture. Red arrow: area of inflammatory infiltrate at the tip of the leaflet which was a feature of two explanted ovine allografts. (b) Upper row of images: sections of the mid-pulmonary artery wall stained with Masson’s trichrome (captured at 5× magnification; scale bars 200 μm). Second row of images: sections of the leaflets stained with Masson’s trichrome (10× magnification; scale bars 100 μm). Third row of images: section stained with von Kossa showing calcium deposits at the proximal suture point of an explanted decellularised porcine pulmonary root at 1 month (20× magnification; scale bar 50 μm), sections stained with Sirius red Miller’s at 3 and 12 months (5× magnification; scale bars 500 μm). The image at the bottom right shows a section of the intimal region in the sinus of Valsalva of an explanted ovine homograft at 12-months stained with H&E (40× magnification; scale bar 20 μm) showing the presence of eosinophils (black arrow), which were a feature of all four explanted ovine allografts in this region. A: adventitia; I: intimal region; M: medial region; MT: Masson’s trichrome; PS: proximal suture; SR: Sirius red; SV, intima of the sinus Valsalva; V: ventricularis. (c) Total numbers of cells in different regions of non-implanted native ovine pulmonary roots, decellularised porcine pulmonary roots following 1, 3 and 12 months implantation and ovine pulmonary root allografts following 12 months implantation in sheep. Data is presented as the mean (n = 4) ± 95% confidence intervals. Data for each region (adventitia, media, intima, leaflet) was analysed by Welch’s Anova followed the Games-Howell post hoc test for significant differences between group means. The bars connect groups which are significantly different (p < 0.05).

Images of representative stained histological sections of the pulmonary roots are shown in Figure 3. The non-implanted native ovine pulmonary roots showed normal pulmonary root tissue histoarchitecture and cellular distribution (Figure 3(a); non-implanted ovine). Upon histological analysis of a third root explanted at 6 months, there was evidence of microbial infection of the tissue. Thus, no further analyses (histology, immunohistochemistry) of any of the roots explanted at 6 months was undertaken. Histological evaluation of the decellularised porcine pulmonary roots explanted at 1, 3 and 12 months showed that they were well integrated with the host tissue and the leaflets were thin and intact (Figure 3(a)). There was a newly formed adventitia from 1 month and a heavy cellular infiltrate surrounding the proximal (Figure 3(a)) and distal suture sites. Cells were dispersed throughout the implanted pulmonary artery wall from 1 month at the highest density in the adventitia and adventitial side of the media with the lowest cell density towards the intimal region (Figure 3(b); MT wall). By 12 months, cells appeared to have penetrated the full thickness of the media of the implanted pulmonary artery wall, however cells were sparser than in the 1- and 3-month explants. There were areas below the intima where no cells were present (Figure 3(b); MT wall). From 1 month, cells were deposited on the ventricular surface and to a lesser extent on the fibrosa of the leaflets. Cellular population of the spongiosa of the leaflets increased at 3 and 12 months (Figure 3(b); MT leaflets). Von Kossa-stained tissue sections showed calcium deposits at the suture sites from 1 month with an absence of calcification throughout the tissues except for one root explanted at 12 months which had microscopic spots of calcium below the intima. The tissues exhibited normal pulmonary root histoarchitecture (Figure 3(b)) with normal collagen and elastin distribution in the wall and the leaflets were thin with the tri-layered structure of ventricularis with elastin present, spongiosa and collagen rich fibrosa layers.

All four ovine allografts explanted at 12 months were sparsely populated with cells. The collagen and elastin fibres in the wall were loosened compared to the non-implanted ovine roots (Figure 3(b); MT wall). The root walls appeared thinner and more elongated and fragile than the explanted decellularised pulmonary artery roots (Figure 3(a)). One leaflet from each of two roots was infiltrated with inflammatory cells (Figure 3(a)). The leaflets of the other two allografts appeared condensed and thinned and were sparsely populated with cells (Figure 3(b); MT leaflet). Small deposits of calcium were also found in sub-intimal areas of the explanted allografts. A striking feature of the explanted ovine allografts was the presence of eosinophilic polymorphonuclear cell foci in the intimal region of the sinus of Valsalva (Figure 3(b); H&E). This was a feature of all four allograft roots.

### Total number of cells populating explanted decellularised porcine pulmonary roots, explanted ovine allografts and non-implanted ovine pulmonary roots

The total number of cells present in different regions of the pulmonary root tissues is presented in Figure 3(c). The non-implanted native ovine pulmonary roots had 1630 ± 259, 2448 ± 515, 2169 ± 627 and 973 ± 100 (mean *n* = 4 ± 95% confidence limits) cells in the adventitia, media, intimal and leaflet regions respectively. There were high numbers of cells present in the adventitia and media regions of the explanted decellularised porcine pulmonary roots after just 1 month of implantation, with lower numbers in the intimal region and leaflets. The numbers remained high in the 3-month explants but then decreased in the adventitia between 3 and 12 months.

Following 1 month’s implantation there were no significant differences between the total number of cells in the adventitia, media or leaflets of the decellularised porcine pulmonary roots compared to the non-implanted ovine controls but the total number of cells in the intima region of the decellularised porcine pulmonary roots was significantly (*p* < 0.05) lower. Following 3 months implantation there were no statistical differences between the total number of cells in the adventitia, media, intima and leaflets of the decellularised porcine pulmonary roots compared to the non-implanted ovine controls. Following 12 months implantation, the total number of cells in all regions of the decellularised pulmonary roots (adventitia, media, intima and leaflets) was significantly lower (p < 0.05) than the non-implanted ovine controls and not significantly different than the explanted ovine allografts.

### Immunohistochemical evaluation of cells populating explanted decellularised porcine pulmonary roots, explanted ovine allografts and non-implanted ovine pulmonary roots

**Figure 4.**
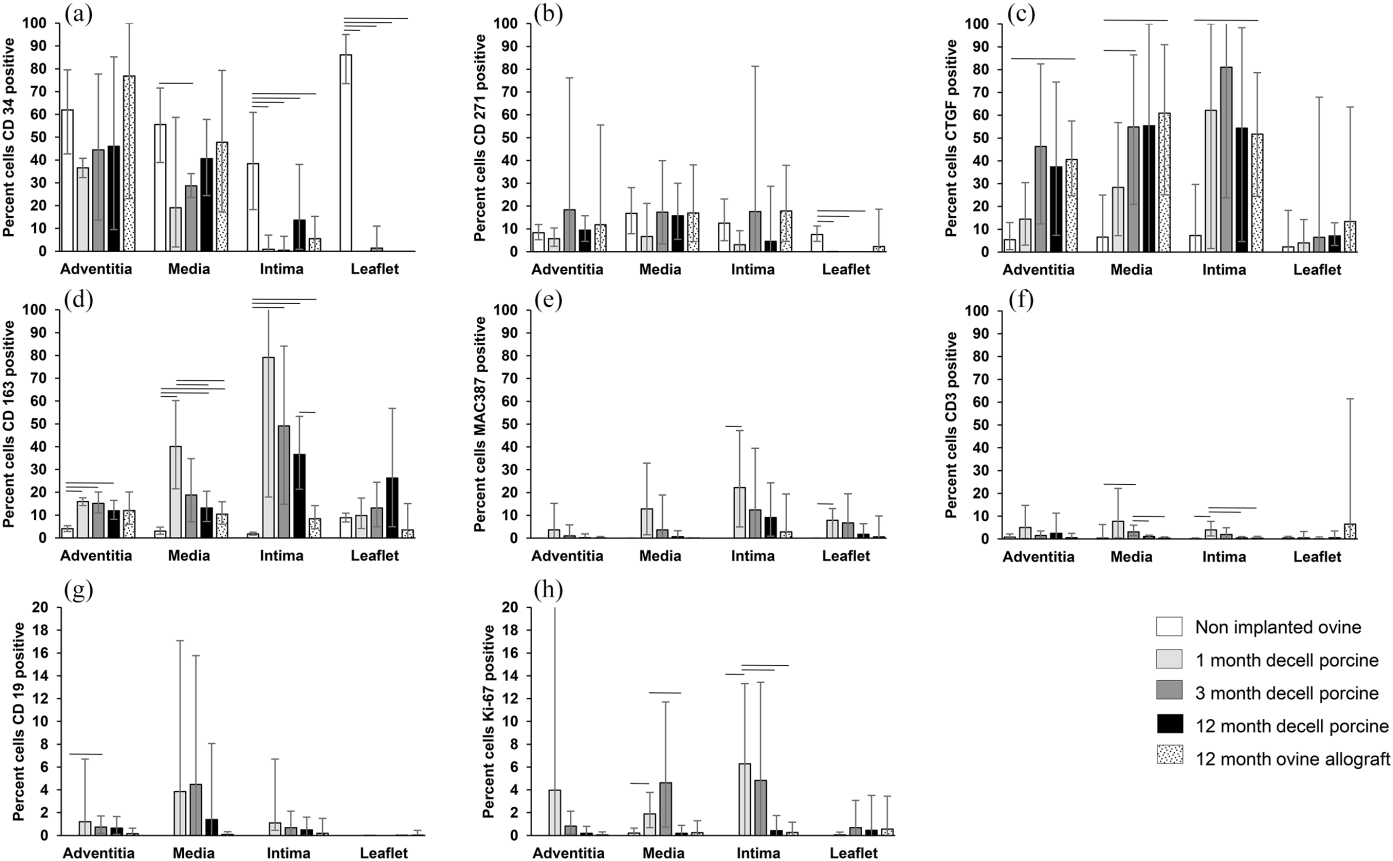
Percentage of total cells that were CD34 (a), CD271 (b), CTGF (c), CD163 (d), MAC 387 (e), CD3 (f), CD19 (g) and Ki67 (h) positive in different regions of non-implanted native ovine pulmonary roots, decellularised porcine pulmonary roots following 1, 3 and 12 months implantation and ovine pulmonary root allografts following 12 months implantation in sheep. Percentage data was arc sin transformed and the mean (n = 4) and 95% confidence limits calculated. Data was back-transformed to percentages for presentation. Arc sin transformed data for each marker for each region (adventitia, media, intima and leaflet) was analysed by Welch’s Anova followed by the Games-Howell post hoc test for significant differences (p < 0.05) between group means. The bars connect groups which are significantly different (p < 0.05). Note that (g and h) are plotted on a smaller scale (0%−20%).

**Figure 5.**
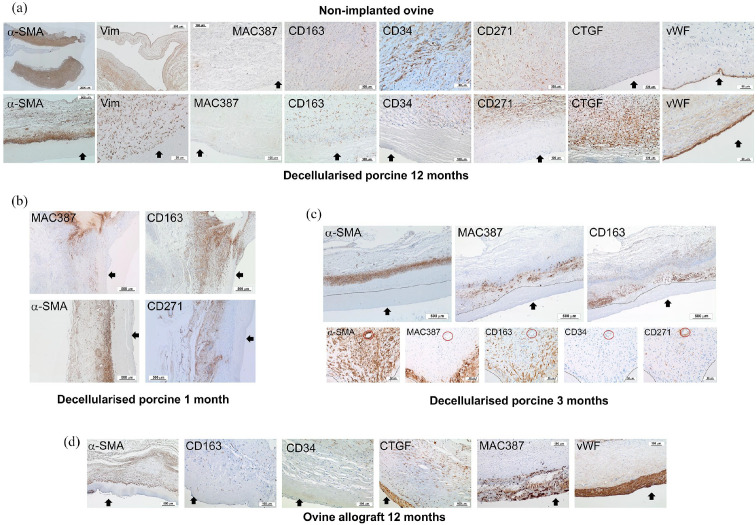
Representative images of sections of explanted pulmonary root wall tissues stained with antibodies to α-SMA, vimentin, MAC 387, CD163, CD34, CD271, vWF and CTGF. (a) Upper panel: native ovine pulmonary artery wall tissues. Images captured at 10× magnification (scale bars 100 μm) unless otherwise stated. α-SMA (scan 2.5× magnification; scale bar 2000 μm), CD34 and vWF (20× magnification; scale bars 50 μm). Lower panel: decellularised porcine pulmonary wall tissues explanted after 12 months. Images captured at 10× magnification (scale bars 100 μm) unless otherwise stated. α-SMA (2.5× magnification; scale bar 500 μm), vimentin and vWF (20× magnification; scale bars 50 μm). Black arrows indicate the intima. (b) Images of the distal decellularised porcine pulmonary artery wall tissues explanted at 1 month. Images captured at 2.5× magnification (scale bars 500 μm). Images show MAC 387+ and CD163+ cells at the interface between cellular and acellular tissue with α-SMA+ and CD271+ cells populating the adventitia and media. Black arrows: intima. (c) Upper panel: images of the same region of central area of a decellularised porcine pulmonary artery wall tissue explanted at 3 months captured at 2.5× magnification (scale bars 500 μm). Images show the ‘front’ (dashed line) of MAC 387+ and CD 163+ cells between the media and intimal regions with α-SMA+ cells in the central media. Black arrows: intima. Lower panel: images of sequential sections of the central area of a decellularised porcine pulmonary artery wall tissue explanted at 3 months captured at 20× magnification (scale bars 50 μm) clearly showing distinct populations of MAC 387+/ CD163^low^; CD163+/ MAC 387+; CD34+ and CD271+ cells. Red circle: vessel present in all images. The dashed lines demarcate cellular and acellular tissue. (d) Ovine allograft pulmonary artery wall tissues explanted at 12 months. Images captured at 10× magnification (scale bars 100 μm) except α-SMA (2.5× magnification; scale bar 500 μm). Images show the presence of α-SMA+, CD163+ and CD34+ cells in the media with an amorphous intimal region and the presence of MAC 387+ cell foci at the intima with high levels of expression of CTGF and vWF. Black arrows: intima.

**Figure 6.**
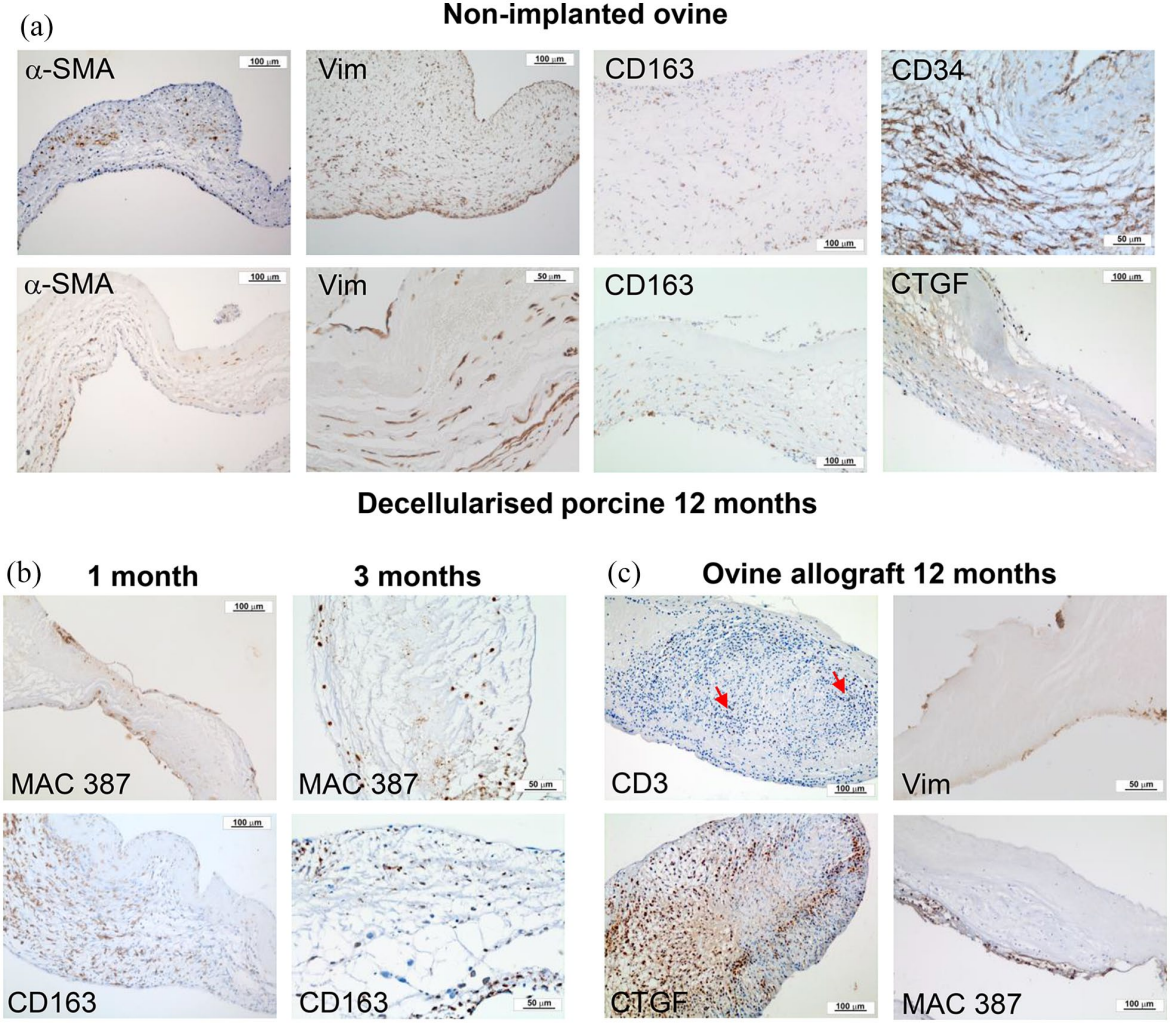
Representative images of sections of explanted pulmonary root leaflet tissues stained with antibodies to α-SMA, vimentin, MAC 387, CD163, CD34 or CTGF. (a) Upper panel: native ovine pulmonary root leaflet tissues. Images captured at 10× magnification (scale bars 100 μm) except CD34 (20× magnification; scale bar 50 μm). Lower panel: decellularised porcine pulmonary wall tissues explanted after 12 months in vivo. Images captured at 10× magnification (scale bars 100 μm) except vimentin (20× magnification; scale bar 50 μm). Images show that the distribution of cells expressing α-SMA, vimentin and CD163 was similar, although sparser, in the decellularised porcine pulmonary root leaflet tissues compared to native ovine tissue, however cells in the decellularised porcine pulmonary root leaflets did not express CD34 and there were high levels of expression of CTGF. (b) Images of explanted decellularised porcine root leaflets stained with antibodies to MAC 387 and CD163 at 1 (images captured at 10× magnification; scale bars 100 μm) and 3 months (images captured at 20× magnification; scale bars 50 μm). At 1 month MAC387+ cells populated the surfaces of the leaflets distally and at 3 months, the body of the leaflets. CD163+ cells populated the basal regions of the leaflets at 1 and 3 months. (c) Images of explanted ovine allograft leaflet tissues at 12 months. Images captured at 10× magnification (scale bars 100 μm) except vimentin (20× magnification; scale bar 50 μm). Images show inflammatory infiltrate with CD3+ and CTGF+ cells (two of four explants) and paucity of vimentin+ cells with MAC 387+ cell infiltrate on leaflet surface (two of four explants).

The number of cells per mm^2^ expressing markers of interest was determined for each tissue region (Supplemental Figure 1). The percentage of the total number of cells in each tissue region expressing each marker is presented in Figure 4. It was not possible to reliably count the numbers of α-SMA and vimentin positive cells in the pulmonary artery wall tissues due to the density of these cells and matrix staining, vWF was used to identify endothelial cells and CD80 positive cells were only sporadically identified. Representative images of sections showing the markers expressed by cells in the pulmonary artery wall and leaflet tissues are shown in Figures 5 and 6 respectively.

**Cellular population of pulmonary artery wall tissues**: The majority of cells in the non-implanted ovine pulmonary root wall tissues were vimentin+ and α-SMA+ (Figure 5(a)). There was a high percentage of CD34+ cells in all regions (38%−62%; Figures 4(a) and 5(a)), indicating CD34+/α-SMA+ cells. CD271+ cells represented a significant (8%−17%) proportion (Figures 4(b) and 5(a)) and a low percentage of the cells expressed CTGF (5%−7%; Figures 4(c) and 5(a)) and CD163 (2%−4%; Figures 4(d) and 5(a)). There were virtually no lymphocytes (CD3, CD19) or MAC 387+ cells (Figure 5(a)) and no proliferating cells (Ki-67) present in the non-implanted ovine pulmonary artery wall tissues (Figure 4(e)–(h)). vWF+ cells were present along the intima (Figure 5(a)).

After 12 months in vivo, the adventitia and media regions of the explanted decellularised porcine pulmonary wall tissues were populated by vimentin+ and α-SMA+ cells (Figure 5(a)) with low numbers in the intimal region which remained relatively devoid of cells. Compared to the native ovine pulmonary root tissues, the percentages of cells expressing CD34 and CD271 in the adventitia and media regions was similar but was lower in the intimal region (Figures 4(a), (b) and 5(a)); the proportion of cells expressing CTGF in all regions of the pulmonary wall tissue was highly variable and not significantly different (Figures 4(c) and 5(a)) and the percentage CD163+ cells in all regions of the explanted decellularised porcine pulmonary root wall were significantly greater than in the native ovine pulmonary root wall (Figures 4(d) and 5(a)). MAC 387+ cells were absent (less than 1%) from the adventitial and medial regions (Figures 4(e) and 5(a)). It was clear that the phenotype of the cells that were present in the intimal region of the 12-month explanted decellularised porcine pulmonary roots (37% CD163+, 9% MAC 387+, 54% CTGF+ and 14% CD34+) was very different from the intimal region of the non-implanted ovine tissue (2% CD163+, 0% MAC 387+, 7% CTGF+, 38% CD34+). vWF+ cells were present at the intima, however vWF expression appeared to be stronger compared to the intimal lining cells of the non-implanted ovine tissue, there was also some staining of the extracellular matrix (Figure 5(a)).

Cellular population of the decellularised porcine pulmonary artery wall tissues appeared to emanate from the adventitia and the media of the host ovine tissue at the proximal and distal suture sites. The ‘pioneering’ cells were predominantly CD163+, however MAC 387+ cells were markedly evident in the process. This is illustrated in the images of the 1-month explants presented in Figure 5(b) with large numbers of CD163+ and MAC 387+ cells present at the interface between cellular and decellularised tissue, particularly around the suture sites and into the intimal region of the wall with α-SMA+, CD34+ and CD271+ cells populating the tissue behind this ‘front’ of largely CD163+ cells. This is supported by the data at 1 month showing that circa 80% of the cells in the intimal region were CD163+ (Figure 4(d)) and 22% MAC 387+ (Figure 4(e)). Moreover, 60% of the cells in the intimal region were CTGF+ (Figure 4(c)) indicating that a proportion of the CD163+ cells were CTGF+. Only circa 2% of the cells were CD34+ or CD271+ (Figure 4(a) and (b)).

At 1 month, the adventitia had been populated with cells expressing vimentin and α-SMA and a new vasa-vasorum had been established (Figure 5(b)). Circa 16% of the cells in the adventitia were CD163+ (significantly greater than in the non-implanted ovine adventitia; Figure 4(d)) and 3% of the cells were MAC 387+ (Figure 4(e)). The percentage of cells expressing CD34, CD271 and CTGF was not significantly different from the percentage in the adventitia of the non-implanted ovine pulmonary root adventitia (Figure 4(a)–(c)). The media was populated with vimentin+ and α-SMA+ cells and a high proportion (40%) of the cells in the media at 4 weeks were CD163+ (40%) which was significantly greater than in the media of the non-implanted ovine tissue (Figure 4(d)) and 13% of the cells were MAC 387+ (Figure 4(e)). Cells expressing CD34 and CD271 represented about 50% of the numbers present in the non-implanted ovine media (Figures 4(a), (b) and 5(b)) and 28% of the cells expressed CTGF (Figure 4(c)).

After 3 months in vivo, the pattern of cellular population of the decellularised porcine pulmonary wall tissues was similar to that seen at 1 month. The images of sections of the mid-pulmonary artery wall tissues stained with antibodies to α-SMA, CD163 and MAC 387 shown in Figure 5(c) clearly indicate the demarcation between cellular and decellularised tissue with the interface demarcated by MAC 387+ and CD163+ cells. In order to determine whether these markers were expressed by different cell types, high power images of sequential sections of the mid-pulmonary artery wall clearly showed two distinct cell types, one which was CD163+/MAC 387− and a second that was MAC 387+ which may have expressed low levels of CD163. Overall, compared to the 1-month explants there were non-significant increases in CD34+ (Figure 4(a)), CD271+ (Figure 4(b)) and CTGF+ (Figure 4(c)) cells and a decrease in CD163+ and MAC 387+ cell numbers in all three tissue regions.

The cells present within the pulmonary artery wall tissues of the ovine allografts explanted at 12 months were predominantly vimentin+ and α-SMA+ and the intimal region had few cells and was amorphous (Figure 5(d)). The percentage of cells in all regions of the wall that were positive for CD34, CD271, CTGF and MAC 387 and percentage of CD163+ cells in the adventitia and media was no different from that of the decellularised porcine pulmonary wall tissues explanted at 12 months. The percentage of CD163+ cells in the intimal region (8%) was, however significantly lower (Figure 4(d)). A key feature was the presence of MAC 387+/ CTGF+/vWF+ cells at the intimal surface (Figure 5(d)) corresponding to the eosinophilic polymorphonuclear cells identified in H&E-stained sections (Figure 3(b); H&E).

CD3+ (Figure 4(f)), CD19+ (Figure 4(g)) and Ki-67+ (Figure 4(h)) cells were not a key feature of the explanted decellularised porcine or ovine allograft pulmonary artery wall tissues, representing less than 8% of cells in any tissues. When present, these cells were in foci around the suture points.

**Cellular population of the leaflet tissues:** α-SMA+ cells were present in the fibrosa of the native ovine leaflet tissues and along the ventricularis, representing circa 30% of the cells (Figure 6(a)). The majority of the interstitial cells in the native non-implanted ovine leaflets were vimentin+ (circa 90%; Figure 6(a)) and CD34+ (86%; Figures 4(a) and 6(a)) with dispersed CD271+ (8%; Figure 4(b)) and CD163+ (9%; Figure 4(d); Figure 6(a)) cells with circa 2% CTGF+ (Figure 4(c)). Most of the interstitial cells in the leaflets of the decellularised porcine pulmonary leaflets explanted at 12 months were vimentin+ (Figure 6(a)) with circa 30% α-SMA+ cells (Figure 6(a)). There were virtually no CD34+ (Figure 4(a)) or CD271+ (Figure 4(b)) cells in the explanted decellularised porcine pulmonary leaflets at 1, 3 or 12 months. The percentage CTGF+ cells in the leaflet tissues was not significantly different to the native non-implanted ovine leaflets at any time point (Figures 4(c) and 6(a)). At 1 month, MAC 387+ cells were present on the leaflet surfaces and the percentage MAC 387+ cells in the leaflets (8%) was significantly greater than in the native non-implanted ovine leaflets. The percentages of MAC 387+ cells reduced over time to 7% at 3 months (Figure 6(b)) and 2% at 12 months (Figure 4(e)), although not significantly. The percentage of leaflet cells expressing CD163 increased over time from 10% at 1 month, where the cells were predominantly in the basal region (Figure 6(b)) to 13% at 3 months and 26% at 12 months (Figures 4(d) and 6(b)). However, neither the absolute number (Supplemental Figure 1) nor the percentage of CD163+ cells was significantly different to the percentage in the native non-implanted ovine leaflets. CD3+, CD19+ and Ki-67+ cells were virtually absent (less than 0.6% of cells) in the explanted decellularised porcine leaflets at any time point (Figure 4(f)–(h)). Two of the explanted ovine allografts at 12 months had inflammatory foci in which CD3+ cells were evident alongside CTGF+ cells (Figure 6(c)) resulting in the large variability in the percentage of cells expressing these markers shown in Figure 4(c) and (f). The leaflets of the other two ovine allografts were largely acellular (Figure 6(c), vimentin) and MAC 387+ cells were identified along the surface of the base of the leaflets (Figure 6(c)).

### Quantitative calcium analysis

The non-implanted native ovine and non-implanted decellularised porcine tissues all had very low levels of calcium, less than 40 ppm wet weight (Table 3). The explanted tissues from the proximal and distal suture sites for the decellularised porcine and ovine allograft pulmonary roots had levels of calcium ranging from 233 ppm (1 month decellularised porcine, proximal suture) to 2813 ppm (12 month decellularised porcine, proximalsuture), and the calcium levels at the suture sites were highly variable. The data for each tissue site was analysed by one-way ANOVA. The calcium levels in the distal suture site for the 12-month explanted decellularised porcine group was significantly greater (p < 0.05) than the same tissue site for the non-implanted ovine and non-implanted decellularised porcine. There were no significant differences in the calcium levels in the tissues from the proximal suture sites of any groups, due to the very high variation in the data.

**Table 3.** Calcium levels in native non-implanted ovine, non-implanted decellularised porcine and explanted decellularised porcine and ovine allograft pulmonary root tissues.

	Distal suture site	Proximal suture site	Distal wall	Proximal wall	Leaflet
Non-implanted ovine	36.6 ± 12.3	27.5 ± 4.6	36.5 ± 6	32.5 ± 5.7	14 ± 8.7
Non-implanted decellularised porcine	12.1 ± 4.3	8.3 ± 1.2	11 ± 2.7	8.6 ± 2	8.7 ± 4.8
Decellularised porcine 1 month	574 ± 349	233 ± 152	90.1 ± 23.9^b^	101 ± 31.4	77.5 ± 16.9
Decellularised porcine 3 months	586 ± 1041	181 ± 53.9	73.7 ± 42	85.9 ± 72.3	51 ± 41
Decellularised porcine 12 months	1450 ± 1492^a,b^	2813 ± 6438	401 ± 57.7^a,b,c,d,e^	389 ± 151a,^b^,^c^,^d^,^e^	278 ± 133^a,b,c,d^
Ovine allograft 12 months	1036 ± 1192	1295 ± 2254	210 ± 74.5^a,b,c,d^	228 ± 45.1^a,b,c,d^	179 ± 99^a,b,c,d^
MSD (*p* < 0.05)	1249	NS	61.7	102.2	100.0

NS: anova showed no significant variation amongst the data.

Data is presented as the mean calcium content in ppm per wet weight (n = 4) ± 95% confidence limits. Data for each tissue site was analysed by one-way analysis of variance, followed by calculation of the minimum significant difference (MSD; p < 0.05). The MSD`s are presented in the bottom row for each type of tissue.

a Significantly greater than non-implanted ovine.

b Significantly greater than non-implanted decellularised porcine.

c Significantly greater than decellularised porcine 1 month.

d Significantly greater than decellularised porcine 3 months.

e Significantly greater than ovine allograft 12 months.

The calcium levels in the explanted decellularised porcine and ovine pulmonary allograft artery wall and leaflet tissues were low and did not exceed 401 ppm wet weight in any of the tissues (Table 3). Nevertheless, analysis of the data revealed that the levels of calcium in the proximal and distal pulmonary artery wall of the explanted decellularised porcine tissue at 12 months was significantly higher (p < 0.05) than the levels in these tissues from the explanted ovine allografts at 12 months. The calcium levels in the proximal and distal artery wall and leaflet tissues of the decellularised porcine roots explanted at 12-month and the ovine allografts explanted at 12 months were significantly greater (p < 0.05) than all other groups tested. The calcium level in the distal decellularised porcine pulmonary artery wall at 1 month was also significantly greater (p < 0.05) than in the non-implanted decellularised porcine distal wall tissue.

### Biomechanical evaluation of ovine and porcine pulmonary root tissues

**Figure 7.**
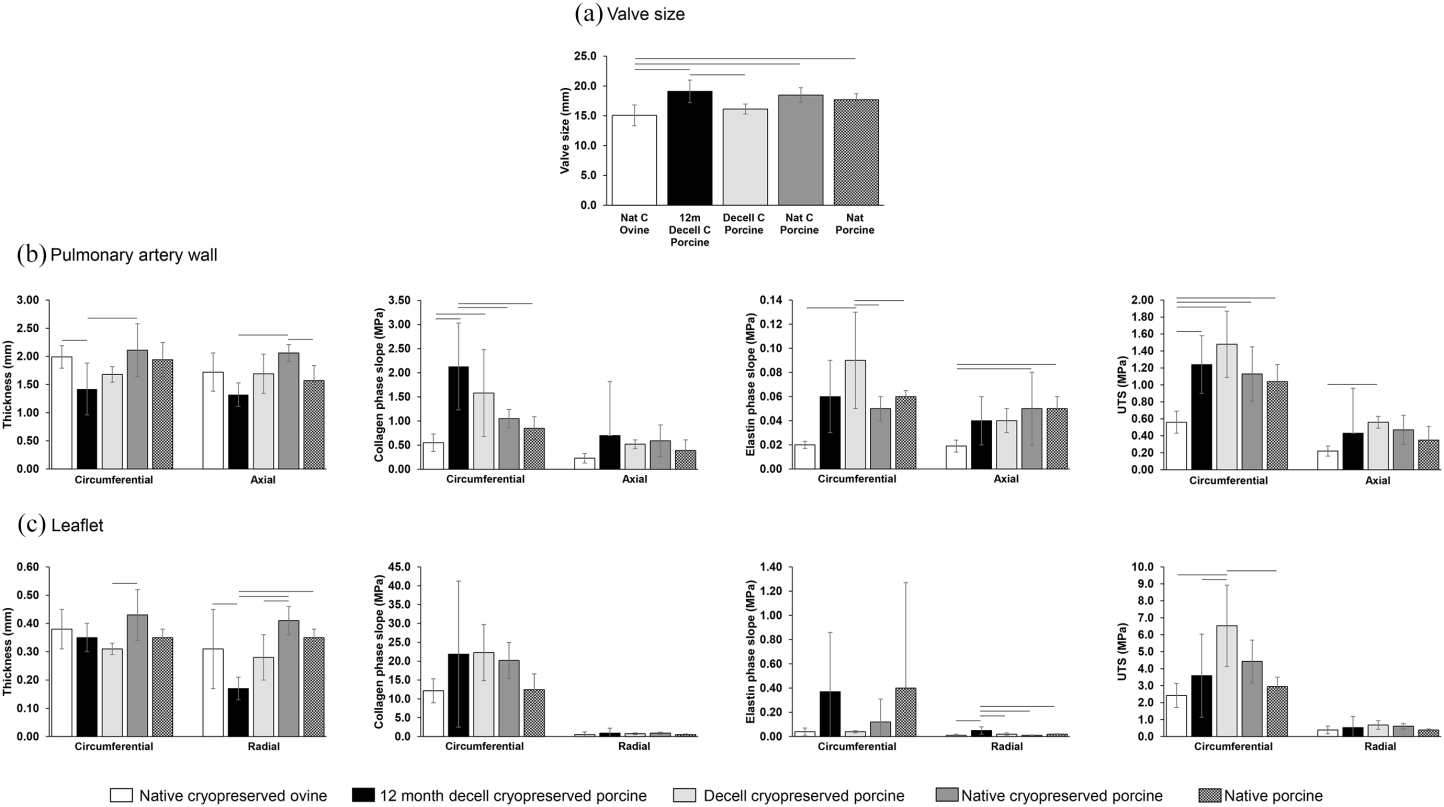
Internal diameter (a) and material properties of the pulmonary artery wall (b) and leaflets (c) of native cryopreserved ovine, native porcine, cryopreserved porcine, decellularised and cryopreserved porcine and explanted (12 months) cryopreserved decellularised porcine pulmonary roots. Data is presented as the mean (n = 6 except n = 4 for 12 months explanted decellularised cryopreserved porcine) ± 95% confidence limits. Data was analysed by ANOVA and Gabriel post hoc test to determine significant differences between groups. The connecting line indicates a significant difference between the two groups (p < 0.05).

The aims of this part of the study were primarily to investigate the material properties of decellularised porcine pulmonary roots following 12 months implantation in sheep compared to pre-implantation (cryopreserved decellularised porcine pulmonary roots) and to cryopreserved native ovine, non-implanted roots. The effect of cryopreservation on the material properties of porcine pulmonary roots and effect of decellularisation and cryopreservation on the material properties of porcine pulmonary roots was also evaluated. The data from the uniaxial testing of the root tissues is presented in Figure 7. Failure of both the wall and leaflet tissue occurred at or near to the centre of the gauge length.

Neither cryopreservation alone nor decellularisation followed by cryopreservation had any major effects on the valve size, collagen phase slope, elastin phase slope and UTS of the pulmonary artery wall and leaflet tissues except for an increase in UTS for cryopreserved decellularised leaflets compared to native porcine leaflets in the circumferential direction (Figure 7(c)). There were some differences in the tissue thickness, with the cryopreserved porcine pulmonary artery wall measured in the axial direction being greater than native porcine roots (Figure 7(b)) and the thickness of the cryopreserved decellularised leaflets reduced in the circumferential and axial directions compared to cryopreserved native porcine pulmonary leaflets (Figure 7(c)).

Regarding the effects of 12-months implantation in sheep on the cryopreserved decellularised porcine roots, the following differences were evident: a pre-implantation valve size of 16.33 mm compared to 19.1 mm when explanted at 12 months (Figure 7(a)), an increase in the elastin phase slope of the leaflets measured in the radial direction (Figure 7(c)) and a decrease in UTS of the leaflets measured in the circumferential direction (Figure 7(c)).

There were some differences in the measured parameters for the 12-month explanted cryopreserved decellularised porcine roots compared to cryopreserved native non-implanted ovine roots. The explanted 12-month cryopreserved decellularised porcine valves (19.1 mm) were larger than cryopreserved native non-implanted ovine valves (15.07 mm); the thickness of the pulmonary artery wall of the cryopreserved decellularised porcine roots explanted from sheep at 12 months (1.42 ± 0.46 mm) was significantly less than the native non-implanted ovine root wall (1.99 ± 0.2 mm; p < 0.05) in the circumferential direction; the pulmonary artery wall of the cryopreserved decellularised porcine roots explanted from sheep at 12 months had a significantly higher collagen phase slope and UTS when compared to the native non-implanted ovine specimens in the circumferential direction (p < 0.05).

The only significant differences between the cryopreserved decellularised porcine leaflet specimens following 12 months implantation in sheep and the native non-implanted ovine leaflet specimens were: the cryopreserved decellularised porcine root leaflets explanted from sheep at 12 months (0.17 ± 0.04 mm) were significantly thinner than the native non-implanted ovine specimens (0.31 ± 0.14 mm; p < 0.05) when measured in the radial direction and had a higher elastin phase slope when tested in the radial direction (0.05 ± 0.03 MPa vs 0.01 ± 0.01 MPa; p < 0.05).

## Discussion

Batches of 18-20 mm decellularised porcine pulmonary roots were produced according to Luo et al.^30^ The roots were oversized for implantation into juvenile sheep to minimise the risk of stenosis due to excess tension in the conduit during the growth of the sheep. Batch analysis showed that the roots were sterile and devoid of cells with total DNA content of less than 18 ng mg wet weight^−1^ for all regions, on the borderline of detection by spectrophotometry, more than fulfilling published criteria for effective decellularisation.^40^

Apart from the roots from the sheep explanted at 6 months which suffered from infective endocarditis the decellularised porcine pulmonary roots showed very good performance in vivo. Quantitative measurements of the velocities of blood flow and pressure gradients across the valves from the Doppler echocardiography revealed no variation between any of the groups of sheep implanted with decellularised porcine or ovine allograft pulmonary roots. All the implanted roots were functioning with blood flow velocities in the normal range and low mean gradients.^41^ The sheep gained weight and the valve size increased over time in vivo, possibly indicating growth potential.^21^ The sheep that received the ovine allograft roots were heavier than the sheep that received the decellularised porcine pulmonary roots. The reasons for this are not clear, since sheep were selected based on age, it may have been seasonal related to weight of fleece, however this did not affect the primary purpose of the study which was to evaluate the in vivo performance of the decellularised porcine pulmonary roots.

Gross analysis of the explanted decellularised porcine pulmonary roots at all the time points revealed that the leaflets were in very good condition being thin, soft and pliable. One third of the explanted decellularised porcine roots exhibited a fenestration in one of the leaflets. Fenestrations in cardiac valve leaflets are a common finding^42^ and this is not believed to compromise valve function. Explanted ovine allografts at 12 months revealed evidence of weakened pulmonary artery walls (two explants) but no other gross abnormalities.

The native ovine pulmonary root tissues demonstrated normal cardiac valve histological features with vWF+ endothelial cells^43^ lining the intima and leaflet cusps and predominantly α-SMA+ vimentin+ cells in the pulmonary wall, with circa 30% of cells in the leaflet tissues α-SMA+ and virtually all cells vimentin+. These findings were consistent with smooth muscle cells in the pulmonary artery wall and quiescent fibroblast-like valve interstitial cells^44^ in the leaflet tissues. CD271 is a broadly accepted marker for multipotential mesenchymal stromal cells (MSC^45^). Between 8% (adventitia and leaflets) to 17% (media) of the cells in the native pulmonary root tissues were CD271+ located around vascular structures, potentially identifying pericytes, but also as discrete irregular shaped stromal cells throughout the tissues. CD34, recognised as a general marker for progenitor cells in a range of different tissues,^46,47^ was expressed by a high percentage of cells ranging from 38% in the intimal region to 86% of the cells in the leaflets. This has not been reported previously in pulmonary valve leaflets of any animals. Interestingly, it has been reported that the stroma of normal human mitral valve cusps is mainly composed of CD34+ cells,^48^ described as fibrocytes. CTGF was expressed by a low percentage of stromal cells with a rounded morphology in all three layers of the pulmonary artery wall (5%−7%) and leaflets (2%).

In order to assess the host response to the implanted decellularised porcine pulmonary roots, markers for macrophages (MAC 387, CD163, CD80), lymphocytes (CD3 for T-cells; CD19 for B-cells; Ki-67 for proliferating cells) were employed. In previous studies in the sheep model, MAC 387, was an appropriate marker for macrophages.^31^ MAC 387 antibodies detect an epitope on migration inhibitory factor related protein (MRP14^49^). MRP 14 is expressed in mononuclear phagocytes and polymorphonuclear leukocytes^50^ and has been used as a marker for recently infiltrating monocytes/macrophages.^51^ Antibodies to CD163 bind to the high affinity scavenger receptor for the haemoglobin-haptoglobin complex expressed by macrophages. In human tissue, CD163 is considered a marker for M2 macrophages.^52–55^ However, markers for macrophages polarisation have not yet been defined in sheep. In sheep lymph nodes, CD163 is expressed by cells in a similar localisation as CD11b+ macrophages.^56^ Here, CD163 was utilised as a marker for tissue macrophages, putatively M2-type macrophages. Antibodies to CD80 bind to the co-stimulatory molecule B7.1 which is expressed by M1 macrophages.^57–60^

Cells expressing CD3, CD19, CD80 and Ki-67 were virtually absent from the native non-implanted ovine pulmonary root tissues. CD163+ cells, however represented 2%−9% of the total cells present and were evenly distributed and of variable morphology. The presence of CD163+ cells in native cardiac valve tissues has not been reported previously. It is likely that these cells represented a tissue resident macrophage population^61–63^ as has been reported in murine arteries^64^ and cardiac myocardium.^65^

There was no evidence a specific immune response to the implanted decellularised porcine pulmonary roots and CD80+ cells were negligible throughout the tissues. There were lymphoid aggregates associated with the suture sites at the earlier time points as has been reported previously.^31^ One month following implantation an extensive vasa-vasorum had been established in the newly formed adventitia rich in blood vessels. vWF+ cells were present on the leaflet surfaces and intima of the pulmonary artery wall at 12 months. Cellular population of the decellularised porcine pulmonary root tissues appeared to be orchestrated by MAC 387+ cells that expressed low amounts of CD163 and CD163+ cells that were MAC 387−. This was particularly evident through the presence of these ‘pioneering’ cells at the interface between the re-populated pulmonary artery wall tissue and tissue that was not populated with cells at 1 and 3 months. The total number and percentage of the total cells that were MAC 387+ or CD163+ was greatest at 1 month in all areas of the pulmonary artery wall tissues and then reduced over time. Stromal cells and progenitor cells (CD34+ and CD271+) appeared to be recruited into the pulmonary artery wall tissues behind the ‘pioneering front’ of macrophages. At 1 and 3 months, a high proportion of the CD34+ cells were associated with microvasculature in the vasa vasorum, around suture sites and in the media of the central pulmonary artery wall, suggesting that these cells were endothelial progenitors perhaps recruited from circulating bone marrow derived progenitors^46^ by factors such as VEGF produced by macrophages. There were also individual elongated CD34+ cells along tissue fibres at all time points, perhaps representing CD34+ stromal cells/fibrocytes.^47^

A high percentage of the cells populating the pulmonary artery wall tissues expressed CTGF particularly in the media and intimal regions at 3 months. The pattern of CTGF expression and high percentage of the total cells expressing this growth factor indicated that it was expressed both by the pioneering macrophages and stromal cells. In the absence of any evidence of tissue fibrosis this may indicate a role in a constructive tissue remodelling process.^65,66^ In the leaflets, MAC 387+ cells were evident on the surface of the leaflets at 1 month with substantial numbers of CD163+ cells in the basal regions. MAC 387+ cell numbers were greatest at 1 month and then declined whereas the percentage of cells that were CD163+ increased from 1 to 12 months, perhaps indicating that recruitment of these cells into the leaflets was delayed compared to recruitment into the pulmonary artery wall.

CD163 was used as a putative marker for M2-type macrophages. The macrophage response to a range of acellular extracellular matrix scaffolds has identified M2 macrophages (CD80-, CD 163+) at the site of constructive remodelling in rats.^32,33^ In our previous studies of decellularised porcine aortic valves in both pigs and sheep, MAC 387+ cells were identified to be involved in the regenerative process.^31^ There were virtually no MAC 387+ cells in the native non-implanted ovine tissues hence the presence of this population of macrophages at an early stage during cellular population of the decellularised porcine pulmonary root tissues suggested that they were recently recruited from blood monocytes.^51^ The large influx of CD163+ cells into the pulmonary artery wall and base of the leaflets observed at 1–3 months, the majority of which clearly did not express MAC 387 or CD34 suggested that these cells may have been recruited from the resident self-renewing macrophage pool in the adjacent ovine tissues. It is hypothesised that the host response to the implanted decellularised porcine tissues was orchestrated by MAC 387+ macrophages recruited from blood monocytes that then recruited tissue resident CD 163+/MAC 387− macrophages in the tissue remodelling response. The data suggested that the constructive remodelling of the pulmonary artery wall tissue was an active process at 12-months and was more advanced in the adventitia and media compared to the intimal region and leaflets. The endothelial cells at the intima of the decellularised pulmonary artery wall at 12-months appeared to express higher levels of vWF compared to the non-implanted ovine. Understanding of whether this was related to ongoing remodelling of the intimal region or developing pathology would require further longer-term studies. Although partially populated with stromal cells, none of the leaflet cells expressed CD34 (expressed by 86% in the native leaflets).

There are numerous studies that have reported upon the partial repopulation of decellularised cardiac valves by endogenous cells in large animal studies including decellularised porcine pulmonary valve xenografts in sheep,^21,41,67–70^ decellularised ovine pulmonary valve allografts in sheep,^37,69–73^ decellularised porcine aortic valve allografts in pigs^1,74,75^ and decellularised porcine aortic valve xenografts in the right ventricular outflow tract of sheep.^31,76,77^ Overall, these studies have reported upon adequate to good in vivo performance of the implanted roots, however most of these studies have evaluated the cellular repopulation of the decellularised valves at a single time point with few studies extended beyond 6 months with limited evaluation of the phenotype of the cells. This is the first study that has quantified and phenotyped the cells repopulating decellularised porcine pulmonary roots in the sheep model at different time points up to 12 months. This is important since the current study indicated that high cell numbers in the decellularised tissues at early time points (1–3 months) may not be sustained over a longer period of time and may reflect the innate host response.

There is only one study^73^ that reported morphometrical analysis of the cellular repopulation of decellularised pulmonary allografts in sheep after 20–21 months in situ. It was shown that the cell density in the pulmonary wall and leaflets represented only 19% and 37% of the cell density in native ovine tissues. The study of Iop et al.^75^ of decellularised aortic valve allografts in pigs at 6 and 15 months evaluated repopulation using a range of histological, immunohistochemical and gene expression analyses. Cells repopulating the valves had phenotypes similar to native valves and importantly, macrophages with an M2 phenotype were highly represented in repopulated valves.

The ovine pulmonary root allograft artery walls did not show a classical T-cell mediated immune rejection response observed for ovine aortic root allografts in our previous studies.^31^ Similar findings have been reported for cryopreserved pulmonary root allografts in sheep at 5 months^68^ post-implantation. The leaflets of two of the four explanted allografts showed thickening and a cell-mediated response with inflammatory infiltrates containing CD3+ T-cells, both of which are known to be responsible for early degeneration.^78^ The leaflets of the other two explanted allografts were sparsely populated with cells. The only significant difference between the cell populations in the decellularised porcine pulmonary explants and ovine allografts at 12 months was a higher percentage of cells expressing CD163 in the intimal region of the decellularised pulmonary artery wall. The most striking feature of the explanted ovine allografts was the presence of eosinophilic polymorphonuclear cell foci in the pulmonary artery wall and intimal region of the sinus/base of leaflets. This was a feature of all four allograft roots revealed by both histology and staining with antibodies to MAC 387. Eosinophils have been previously implicated in some cases of acute allograft rejection responses (reviewed in Long et al.^79^). Moreover, the media of the pulmonary artery wall ovine allograft explants appeared to be stretched and delaminating. This was consistent with the findings of a rupture in one of the explants and an aneurysm at the site of the sinus Valsalva in another.

All explanted pulmonary roots had high levels of calcium in the tissue at both the proximal and distal suture sites as has been reported previously.^31,37,72,75^ The calcium content of the explanted cryopreserved ovine allograft tissues after 12 months was low; 210–228 ppm (mg kg^−1^ wet weight) in the pulmonary wall tissues and 179 ppm (mg kg^−1^ wet weight) in the leaflets. The lack of calcification of the ovine pulmonary allografts may be associated with the absence of an overt T-cell mediated immune response. Previous studies of cryopreserved ovine pulmonary root allografts in the sheep model have shown either calcification of the pulmonary artery wall^37,72^ or calcium levels being extremely low.^68^ Hence, there may be differences between the performance of cryopreserved pulmonary allografts in sheep dependent upon differences in sheep breed and/or method of cryopreservation.

The calcium content of the 12-month explanted decellularised porcine pulmonary root wall was approximately 400 ppm (mg kg^−1^ wet weight) whilst the calcium content of the leaflets was circa 300 ppm (mg kg^−1^ wet weight). Whilst these values were low, and there was no significant difference in the levels of calcium in the leaflets compared to the 12-month explanted cryopreserved ovine allografts, the calcium levels in the pulmonary artery wall were significantly greater. This may have been due to differences in the length of the explanted pulmonary artery root walls. The 12-month explanted ovine allograft roots (5.4 ± 2.8 cm) were longer than the 12-month explanted decellularised porcine pulmonary roots (2.9 ± 1.0 cm), making it more likely that there was overlap between the samples taken for calcium analysis between the proximal and distal suture sites and proximal and distal wall samples for the shorter decellularised porcine compared to the longer ovine allograft wall tissues.

Much of the literature on the performance of decellularised allogeneic or xenogeneic pulmonary roots in the sheep model has only reported histological or radiological qualitative observations.^21,37,41,67,69,72,75^ Della Barbera et al.^73^ reported calcium levels of 1.667 mg g^−1^ (dry weight; circa 416 ppm wet weight assuming 75% water content) in the wall and 0.597 mg g^−1^ (circa 149 ppm wet weight) in the leaflets for native ovine pulmonary roots and 2.17 mg g^−1^ (circa 543 ppm wet weight) in the pulmonary wall and 2.77 mg g^−1^ (circa 693 ppm wet weight) in the leaflets of explanted decellularised pulmonary allografts at 14–21 months. Acharya et al.^80^ measured calcium in native ovine pulmonary valve leaflets at 0.37 μg mg^−1^ (dry weight; circa 93 ppm wet weight). These calcium levels were higher than the levels of calcium found in the current study. A likely explanation is that, in the present study the tissues used for calcium analysis had been fixed in formalin and transported in ethanol prior to analysis potentially removing calcium from the extracellular fluid.

The levels of calcium quantified in the explanted decellularised porcine pulmonary valve tissues were, however, low and not detected in the tissues (away from the suture points) by gross analysis, functional performance or Von Kossa staining of histological sections, except for one root explanted at 12 months which had microscopic spots of calcium in the elastic lamella of the wall. It was therefore concluded that the calcium levels in the explanted decellularised pulmonary roots after 12 months implantation in sheep, were not of major concern for future clinical translation.

Biomechanical evaluation was carried out to determine the effects of decellularisation and cryopreservation on the material properties of porcine pulmonary roots and the material properties of these roots were evaluated following 12 months implantation in sheep. It was clearly shown that the pre-implantation processing steps that the porcine pulmonary valve tissue was subjected to (cryopreservation and decellularisation) did not alter tissue integrity to the extent that it would have been likely to impair biomechanical function. For the decellularised porcine pulmonary roots implanted for 12 months in sheep, the material properties of the wall were similar to non-implanted decellularised porcine pulmonary roots. However, for the leaflets, the UTS in the circumferential direction was significantly reduced and the elastin phase stiffness in the radial direction was significantly increased. There were however no other significant differences between the decellularised porcine pulmonary roots implanted for 12 months and those not implanted. The material properties of the cryopreserved native ovine pulmonary root showed a trend of lower stiffness and strength for all wall and leaflet specimens investigated when compared to any of the equivalent porcine groups.

The explanted cryopreserved decellularised porcine pulmonary roots at 12 months were significantly larger in size compared to the cryopreserved native non-implanted ovine valves, perhaps as a result of oversizing these roots at the time of implantation. These results should be interpreted with caution because of differences in root sizes and species.

There are several limitations to this study. The low sample size at each time point, limited the statistical power, and there was a high level of biological variation within the groups. The loss of histological and immunohistochemical data at the 6-month time point was a limitation but this did not impact the outcome of the study. Although an extensive number of antibodies were used in the immunohistochemical analysis, this could have been extended to additional markers. The native ovine pulmonary roots from 15-month-old sheep used for comparison in the material properties and morphometric analyses were significantly smaller than the 12-month explanted cryopreserved decellularised porcine pulmonary roots. The lack of native ovine pulmonary roots explanted at 12 months for comparison of material properties was also a limitation.

In conclusion, the low concentration SDS decellularised porcine pulmonary roots showed good functional performance in the RVOT of sheep over a 12-month period. The decellularised pulmonary root tissues were repopulated with ovine cells of the appropriate phenotype in a process orchestrated by macrophages of an M2 phenotype, highlighting that these cells may be important in the constructive tissue remodelling of cardiac root tissues. Following 12 months in vivo, however, the extent of cellular repopulation was less than 50% of the cellular population in native ovine valved conduit tissues of a similar age. Longer term studies would be required to determine whether the stromal cell densities reached those of native sheep pulmonary valve tissues. Importantly, the fact that the leaflets, the primary location of valve dysfunction, were populated with stromal cells, indicated the potential for matrix repair and remodelling.

## Supplemental Material

sj-docx-1-tej-10.1177_20417314221102680 – Supplemental material for Repopulation of decellularised porcine pulmonary valves in the right ventricular outflow tract of sheep: Role of macrophages Click here for additional data file. Supplemental material, sj-docx-1-tej-10.1177_20417314221102680 for Repopulation of decellularised porcine pulmonary valves in the right ventricular outflow tract of sheep: Role of macrophages by Tayyebeh Vafaee, Fiona Walker, Dan Thomas, João Gabriel Roderjan, Sergio Veiga Lopes, Francisco DA da Costa, Amisha Desai, Paul Rooney, Louise M Jennings, John Fisher, Helen E Berry and Eileen Ingham in Journal of Tissue Engineering

sj-tif-2-tej-10.1177_20417314221102680 – Supplemental material for Repopulation of decellularised porcine pulmonary valves in the right ventricular outflow tract of sheep: Role of macrophages Click here for additional data file. Supplemental material, sj-tif-2-tej-10.1177_20417314221102680 for Repopulation of decellularised porcine pulmonary valves in the right ventricular outflow tract of sheep: Role of macrophages by Tayyebeh Vafaee, Fiona Walker, Dan Thomas, João Gabriel Roderjan, Sergio Veiga Lopes, Francisco DA da Costa, Amisha Desai, Paul Rooney, Louise M Jennings, John Fisher, Helen E Berry and Eileen Ingham in Journal of Tissue Engineering
