# Supplementary material for: Repopulation of decellularised porcine pulmonary valves in the right ventricular outflow tract of sheep: Role of macrophages
Source: J Tissue Eng. 2022 Jun 28;13:20417314221102680. doi: 10.1177/20417314221102680 (PMC9243591; doi:10.1177/20417314221102680)
Supplement: sj-docx-1-tej-10.1177_20417314221102680 – Supplemental material for Repopulation of decellularised porcine pulmonary valves in the right ventricular outflow tract of sheep: Role of macrophages [file sj-docx-1-tej-10.1177_20417314221102680.docx]

**Supplementary information**

**Repopulation of decellularised porcine pulmonary valves in the right ventricular outflow tract of sheep: role of macrophages.**

Tayyebeh Vafaee, Fiona Walker, Dan Thomas, João Gabriel Roderjan , Sergio Veiga Lopes, Francisco DA da Costa, Amisha Desai, Paul Rooney, Louise M Jennings, John Fisher, Helen E Berry, Eileen Ingham.

**Supplementary Figure (1)** **Total number of** **cells that were CD34 (A), CD271 (B), CTGF (C), CD163 (D), MAC (E), CD3 (F), CD19 (G) and Ki67 (H) positive in different regions of non-implanted native ovine pulmonary roots, decellularised porcine pulmonary roots following 1,3 and 12 months implantation and ovine pulmonary root allografts following 12 months implantation in sheep.** Data is presented as the mean (n=4) ± 95% confidence intervals. Data for each region (adventitia, media, intima and leaflet) for each marker was analysed by Welch`s Anova followed by the Games-Howell post-hoc test for significant differences (p<0.05) between group means. The bars connect groups which are significantly different (p< 0.05).
