# Supplementary figures and images for: Repopulation of decellularised porcine pulmonary valves in the right ventricular outflow tract of sheep: Role of macrophages
Source: J Tissue Eng. 2022 Jun 28;13:20417314221102680. doi: 10.1177/20417314221102680 (PMC9243591; doi:10.1177/20417314221102680)

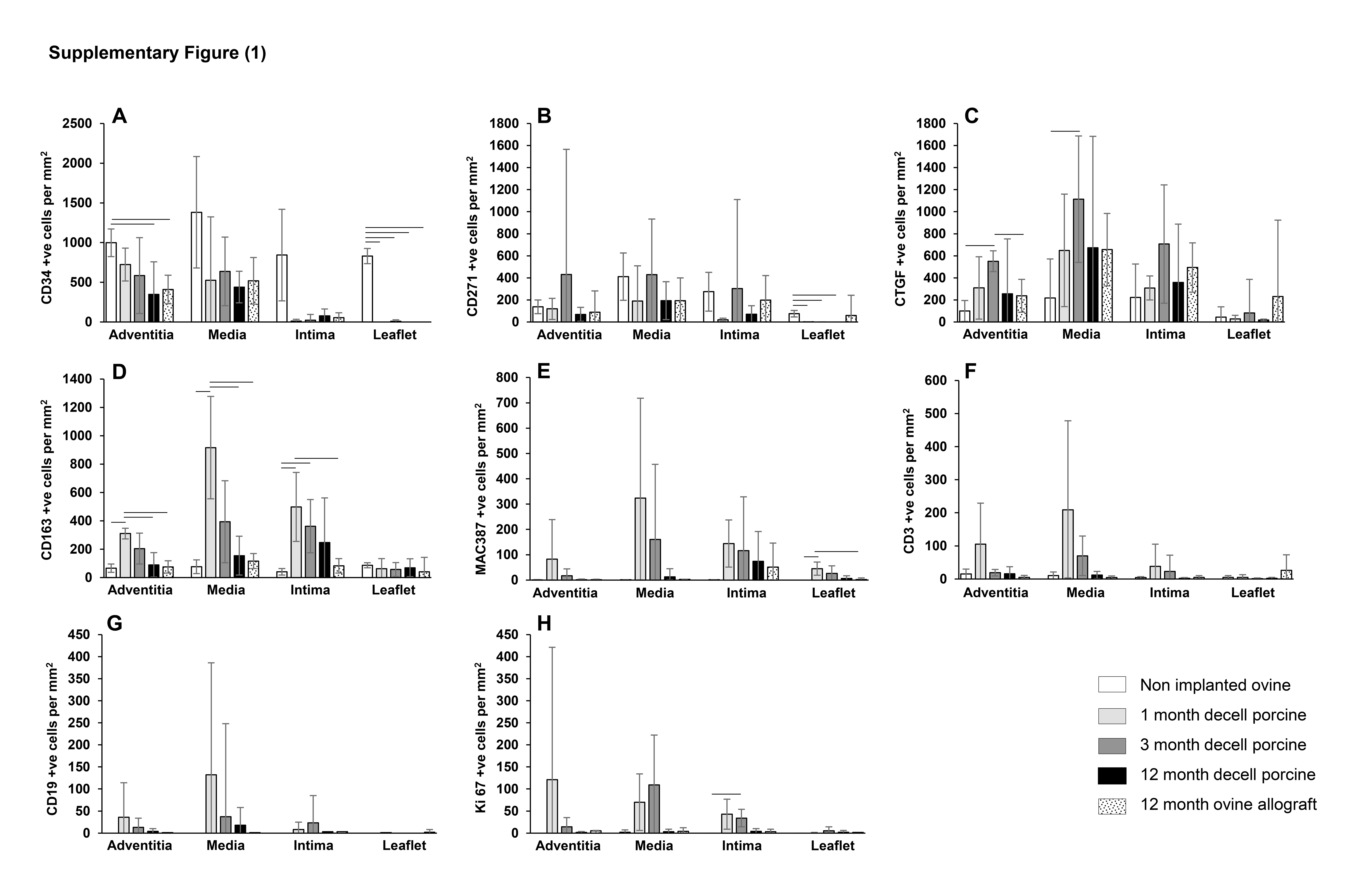

Supplement: sj-tif-2-tej-10.1177_20417314221102680 – Supplemental material for Repopulation of decellularised porcine pulmonary valves in the right ventricular outflow tract of sheep: Role of macrophages [file sj-tif-2-tej-10.1177_20417314221102680.tif]
